# Redox‐Responsive Dual Drug Delivery Nanosystem Suppresses Cancer Repopulation by Abrogating Doxorubicin‐Promoted Cancer Stemness, Metastasis, and Drug Resistance

**DOI:** 10.1002/advs.201801987

**Published:** 2019-02-04

**Authors:** Jia Liu, Bingcheng Chang, Qilin Li, Luming Xu, Xingxin Liu, Guobin Wang, Zheng Wang, Lin Wang

**Affiliations:** ^1^ Research Center for Tissue Engineering and Regenerative Medicine Union Hospital Tongji Medical College Huazhong University of Science and Technology Wuhan 430022 China; ^2^ Department of Clinical Laboratory Union Hospital Tongji Medical College Huazhong University of Science and Technology Wuhan 430022 China; ^3^ Department of Gastrointestinal Surgery Union Hospital Tongji Medical College Huazhong University of Science and Technology Wuhan 430022 China

**Keywords:** cancer repopulation, cancer stemness, celecoxib, chemotherapy, prostaglandin E2

## Abstract

Chemotherapy is a major therapeutic option for cancer patients. However, its effectiveness is challenged by chemodrugs' intrinsic pathological interactions with residual cancer cells. While inducing cancer cell death, chemodrugs enhance cancer stemness, invasiveness, and drug resistance of remaining cancer cells through upregulating cyclooxygenase‐2/prostaglandin‐E2 (COX‐2/PGE_2_) signaling, therefore facilitating cancer repopulation and relapse. Toward tumor eradication, it is necessary to improve chemotherapy by abrogating these chemotherapy‐induced effects. Herein, redox‐responsive, celecoxib‐modified mesoporous silica nanoparticles with poly(β‐cyclodextrin) wrapping (MSCPs) for sealing doxorubicin (DOX) are synthesized. Celecoxib, an FDA‐approved COX‐2 inhibitor, is employed as a structural and functional element to confer MSCPs with redox‐responsiveness and COX‐2/PGE_2_ inhibitory activity. MSCPs efficiently codeliver DOX and celecoxib into the tumor location, minimizing systemic toxicity. Importantly, through blocking chemotherapy‐activated COX‐2/PGE_2_ signaling, MSCPs drastically enhance DOX's antitumor activity by suppressing enhancement of cancer stemness and invasiveness as well as drug resistance induced by DOX‐based chemotherapy in vitro. This is also remarkably achieved in three preclinical tumor models in vivo. DOX‐loaded MSCPs effectively inhibit tumor repopulation by blocking COX‐2/PGE_2_ signaling, which eliminates DOX‐induced expansion of cancer stem‐like cells, distant metastasis, and acquired drug resistance. Thus, this drug delivery nanosystem is capable of effectively suppressing tumor repopulation and has potential clinical translational value.

## 1 Introduction

Chemotherapy remains a major therapeutic approach for clinic oncotherapy. Although cytotoxic chemotherapeutics brutally kill cancer cells, cancer relapse still nearly inevitably occurs even after removal of tumor mass, which accounts for 90% of cancer death.^1^ An increasing number of studies suggest that cancer relapse after chemotherapy treatment is partly attributable to chemodrugs' effects on promoting stemness^2^ and metastasis[[qv: 2f,h]] of residual cancer cells, and enhancing their drug resistance capability.[[qv: 2b,3]] These effects derived from chemotherapy act as a “backdoor” for cancer cells to re‐thrive.[[qv: 3a,4]] However, such “backdoor” is often neglected in our day‐to‐day oncological clinical practice, largely due to lack of clinically approved agents to effectively block this “backdoor” toward reducing cancer repopulation.

A key mechanism lately discovered underlying chemodrugs' (e.g., gemcitabine, cisplatin and doxorubicin) boosting cancer stemness, metastasis and drug resistance is that these chemodrugs can upregulate cyclooxygenase‐2 (COX‐2), a critical enzyme for synthesizing prostaglandin E2 (PGE_2_), in cancer cells and promote PGE_2_ release.[[qv: 2b,5]] This released PGE_2_ in tumor microenvironment not only activates existing quiescent cancer stem cells (CSC) to proliferate,[[qv: 2b,6]] contributing to tumor recurrence,[[qv: 2b]] but also drives the identity conversion of non‐CSCs tumor cells to be CSC‐like cells.^7^ Meanwhile, PGE_2_ reportedly promotes cancer cell dissemination by mobilizing them via several molecular pathways key to cell migration and invasion.^7^, ^8^ Further, PGE_2_ enhances expression of P‐glycoprotein (P‐gp), a prominent protein mediating cancer multidrug resistance.^9^ High‐level PGE_2_ reportedly contributes to drug resistance in various cancers, including liver cancer^10^ and breast carcinoma,^11^ and significantly impacts clinical outcomes.^12^ Thus, an effective chemotherapy strategy aiming to eradicate a tumor should be capable of abrogating COX‐2/PGE_2_ axis that opens up the “backdoor” facilitating cancer repopulation.

As a specific COX‐2 inhibitor approved by FDA, celecoxib reportedly reverses PGE_2_‐mediated CSCs expansion[[qv: 2b,3b,13]] and metastasis,[[qv: 6c,7]] and dampens P‐gp‐dependent drug resistance induced by chemotherapy,[[qv: 2b,3b,10a,11b,14]] thereby presumably suppressing cancer repopulation. This notion is supported by several observations that celecoxib helped reduce cancer incidence and pre‐cancerous lesion occurrence in high‐risk populations (for instance, colon and skin cancer).^15^ However, given celecoxib's poor solubility and low bioavailability as well as limited tumor infiltration capability,^16^ celecoxib has to be orally administered in a high dose, which can lead to severe cardiovascular events[[qv: 15c,17]] that resulted in termination of two government‐sponsored clinical trials.^18^

**Figure 1:**
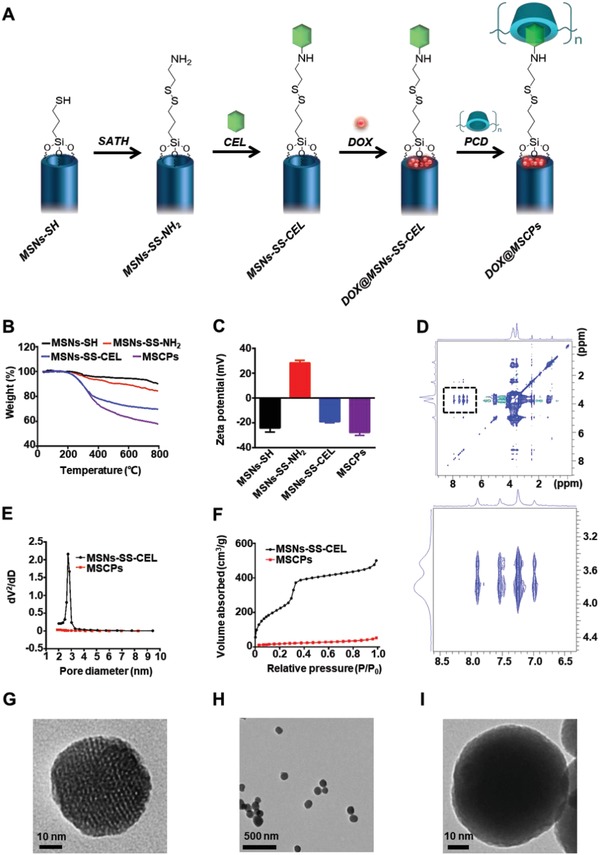
Preparation and characterizations of MSNs. A) Schematics showing synthesis process of DOX‐loaded, redox‐responsive, celecoxib‐modified mesoporous silica nanoparticles (DOX@MSCPs). B) TGA and C) zeta potential of MSNs‐SH, MSNs‐SS‐NH_2_, MSNs‐SS‐CEL, and MSCPs (*n* = 3). Data shown as mean ± SD. D) 2D ^1^H NOESY spectrum of PCD/CEL in D_2_O. E) Pore size distributions and F) N_2_ adsorption isotherms of MSNs‐SS‐CEL and MSCPs. TEM images of G) MSNs‐SS‐CEL and H,I) MSCPs.

Aiming at suppressing cancer relapse with a goal of tumor eradication, we hypothesized that a nanosized drug carrier system co‐delivering chemodrugs and hydrophobic celecoxib to tumor sites would be a promising approach for improving chemotherapy while eliminating “backdoor” effects. Thus, we proposed to design a multifunctional drug delivery nanosystem (**Figure** 1A) using 1) mesoporous silica nanoparticles (MSNs) as the carrier base to load with chemodrugs (doxorubicin, DOX), 2) poly(β‐cyclodextrin) (PCD) as a functional gatekeeper topologically like a corona wrapping MSNs, and 3) celecoxib as a component of the linker connecting MSNs' surface with PCD gatekeeper. In this design, the linker was a redox‐responsive disulfide linkage where celecoxib molecules were a structural component and would be released away from the MSNs' surface in response to reducing microenvironment. Of note, celecoxib molecules were chemically modified to attach outward to cyclodextrin units of PCD via host‐guest supramolecular interactions. Therefore, cleavage of this disulfide linker should allow dual release of celecoxib and DOX.

**Scheme 1:**
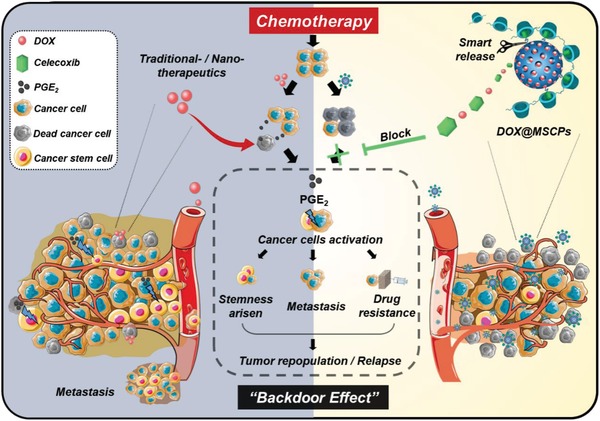
Schematic illustration of the “backdoor” of chemotherapy (left) and therapeutic strategy of DOX@MSCPs (right).

Here, we reported that we synthesized such redox‐responsive celecoxib‐modified MSNs via disulfide linkages (MSNs‐SS‐CEL), and employed PCD as the gatekeeper for sealing DOX within MSNs' pores, which thus gave rise to the desired nanosystem (MSCPs). MSCPs nanosystem could effectively deliver these two molecules into tumor local. By blocking the COX‐2/PGE_2_ axis, MSCPs increased the sensitivity of drug‐resistant cancer cells to DOX and abrogated the DOX‐induced enhancement on cancer stemness, metastasis and P‐gp expression (**Scheme** 1). MSCPs also achieved these effects in vivo in three preclinical animal models (human liver cancer cells xenografted primary tumor model, murine metastatic breast cancer orthotopic model, and multiround‐chemotherapy treated breast cancer orthotopic model). Thus, MSCPs containing celecoxib moieties are a promising drug delivery nanosystem toward tumor eradication.

## 2 Results and Discussion

### 2.1 Synthesis and Characterization of MSCPs

The redox‐sensitive MSNs‐based drug delivery system with COX‐2 inhibition activity was fabricated in four steps (Figure 1A): 1) through hydrolysis of TEOS as our previously reported,^19^ MSNs (MCM‐41 type) were functionalized with thiol (‐SH) groups to generate MSNs‐SH; 2) MSNs‐SH were then reacted with S‐(2‐aminoethylthio)‐2‐thiopyridine hydrochloride (SATH) to obtain amine modified MSNs with disulfide linkages (MSNs‐SS‐NH_2_); 3) amine groups of MSNs‐SS‐NH_2_ were covalently conjugated with celecoxib succinamidic acid (a celecoxib prodrug, CEL) (Figure S1, Supporting Information) to prepare MSNs‐SS‐CEL; (4) after DOX was loaded into MSNs‐SS‐CEL, the nanoparticles' celecoxib moieties were capped by cyclodextrin units of PCD through host‐guest interactions, consequently yielding PCD‐capped, DOX‐loaded MSNs‐SS‐CEL nanoparticles (DOX@MSNs‐SS‐CEL@PCD), which were termed as DOX@MSCPs for simplicity (Figure 1A). The MSNs without DOX (MSNs‐SS‐CEL@PCD) were also synthesized and denoted as MSCPs.

The synthetic processes of MSNs‐SS‐CEL@PCD were confirmed by a stepwise increased weight loss across the intermediate nanoparticles (MSNs‐SH, 10%; MSNs‐SS‐NH_2_, 16%; MSNs‐SS‐CEL, 31%; MSCPs, 43%) in thermal gravimetric analysis (TGA), and the changes in zeta‐potential of MSNs (MSNs‐SH, −23 mV; MSNs‐SS‐NH_2_, +28 mV; MSNs‐SS‐CEL, −19 mV; MSCPs, −28 mV) (Figure 1B,C). The host‐guest interactions between PCD and celecoxib succinamidic acid were investigated using 2D ^1^H nuclear Overhauser effect spectroscopy (NOESY) in D_2_O. The NOE cross peaks between the inner protons of CD units (3.5 to 4.2 ppm) and the protons of the p‐tolyl group on celecoxib (6.8–8.0 ppm) were clearly detected (Figure 1D), suggesting that PCD attaches to celecoxib moieties on the surface of MSNs‐SS‐CEL. The synthesized MSNs‐SS‐CEL had spherical morphology with well‐defined mesostructure (Figure 1E,G). After being capped with PCD, MSNs' mesostructure turned misty, while the spherical shape was maintained (Figure 1H,I). Pores of the nanoparticles became undetectable from 2.7 nm in MSNs‐SS‐CEL, and surface area significantly dropped from 501 to 50 m^2^ g^−1^ (Figure 1E,F). Together, these results indicate that celecoxib‐grafted MSNs (MSNs‐SS‐CEL) are successfully synthesized and PCD effectively wraps the surface of MSNs, thus giving rise to the desired nanocarrier system (MSCPs).

Additionally, to generate a control for precisely studying celecoxib's specific effects, we grafted MSNs with phenyl‐groups (MSNs‐SS‐Bz@PCD) in parallel because of the structural similarity between phenyl groups and celecoxib (Scheme S1; Figure S2A,B, Supporting Information).^19^ We termed this control nanosystem “MSBPs.”

### 2.2 Acidity‐Promoted, Redox‐Responsive Dual Drug Release Behaviors, and COX‐2 Inhibitory Property of MSCPs

**Figure 2:**
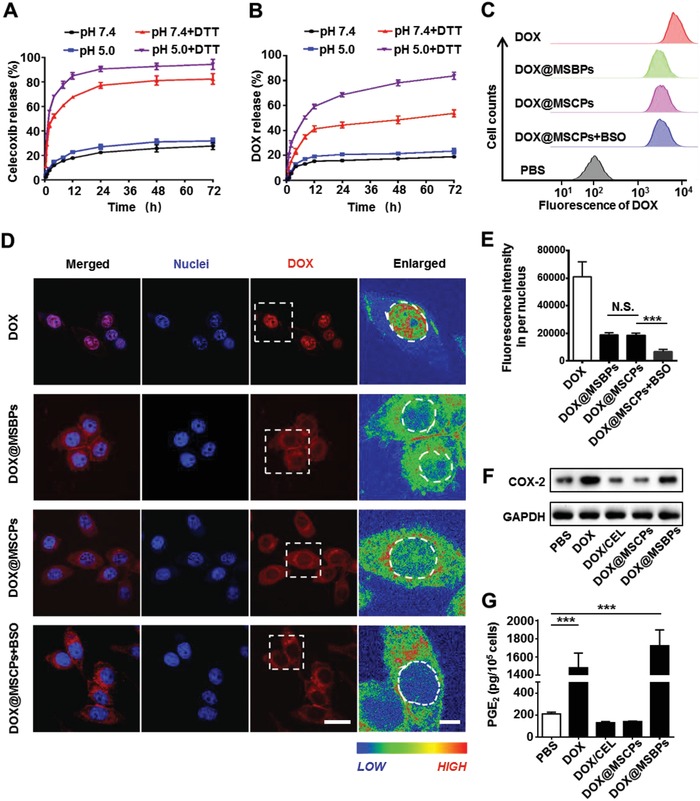
Drug release behaviors and the COX‐2 inhibition effect of DOX@MSCPs in vitro. A,B) In vitro release profiles of DOX and celecoxib from DOX@MSCPs in PBS (pH 7.4, with or without 10 × 10^−3^ m DTT) or acetate buffer (pH 5.0, with or without 10 × 10^−3^ m DTT). C) Flow cytometry analysis of HepG2 cells incubated with DOX or DOX‐loaded nanoparticles (4 µg mL^−1^ of DOX) for 2 h. D) Cellular uptake and intracellular DOX release in HepG2 cells. The fluorescence intensity was transformed to enlarged spectrum representation. Left scale bar, 10 µm. Right scale bar, 3 µm. E) Quantitative analysis of DOX fluorescence intensity in the nuclei of HepG2 cells (*n* = 100). F) Western blot analysis of relative COX‐2 protein levels of HepG2 cells after being treated with DOX, DOX/CEL (DOX and celecoxib succinamidic acid), or DOX‐loaded nanoparticles for 36 h. G) PGE_2_ released from HepG2 cells after being treated with DOX, DOX/CEL, or DOX‐loaded nanoparticles for 36 h. PGE_2_ contents were normalized to live cell numbers and indicated as pg/10^5^ cells (*n* = 3). Data shown as mean ± SD, ***p < 0.001, N.S., not significant.

The loading amounts of DOX and celecoxib in DOX@MSCPs were determined, 13.1% and 15.9%, respectively. We next examined the release behaviors of DOX and celecoxib from this nanosystem. In pH 7.4 and 5.0 conditions (mimicking blood circulation and lysosomal microenvironment, respectively), the limited release of celecoxib (<30%) and DOX (<20%) from MSCPs was observed, indicating that DOX@MSCPs effectively encapsulate the cargos preventing premature leakage. Nevertheless, the addition of DTT (10 × 10^−3^ m) that can cleave disulfide bonds drastically promoted the release of DOX and celecoxib at pH 7.4, and even more effectively at pH 5.0 (**Figure** 2A,B). This was likely because 1) the acidity accelerated the hydrolysis of the sulfanilamide linkage between celecoxib and succinic acid; 2) DOX was more hydrophilic at pH 5.0 than at pH 7.4, resulting in a faster diffusion once MSNs' coating was cracked. These results suggest that MSCPs securely encapsulate DOX by PCD corona, while corelease DOX and celecoxib in response to redox environment; and this release is more effective when this environment is of acidity.

Next, cellular uptake efficiency and intracellular DOX release mechanisms of DOX@MSCPs were studied in human hepatocellular carcinoma cell line (HepG2). DOX@MSCPs were engulfed by cells into endo‐ and lysosomal compartments (Figure S3, Supporting Information), an acidic and reductive microenvironment (pH 4.5–6.0; containing 2–10 × 10^−3^ m glutathione). Buthionine sulphoximine (BSO) capable of downregulating intracellular glutathione level by inhibiting γ‐glutamylcysteine synthetase was employed to examine redox‐responsive dual release of DOX and celecoxib. After a 2 h incubation, the similar amounts of DOX@MSCPs and DOX@MSBPs were internalized to perinuclear regions (Figure 2C,D) with also the similar amounts of DOX accumulated (23.8% and 24.1%, respectively) in the nuclei (Figure 2E). Notably, the addition of BSO led to a 63% reduction on DOX nuclear accumulation in DOX@MSCPs treated cells (Figure 2E). This was not due to BSO interfering cellular uptake of the nanoparticles (Figure 2C). Thus, these results demonstrate that DOX intracellular release from MSCPs is redox‐responsive and glutathione‐dependent.

MSCPs were designed to release celecoxib derivative from the surface of MSNs upon cleavage of disulfide bonds. We then tested MSCPs' inhibitory effects on COX‐2 in HepG2 cells. Free DOX and DOX@MSBPs treatments significantly upregulated COX‐2 protein levels and PGE_2_ production (Figure 2F,G and Figure S4, Supporting Information), revealing DOX's promoting effects on COX‐2 expression and PGE_2_ generation, consistent with the notion that chemodrugs are capable of activating COX‐2/PGE_2_ axis.[[qv: 2b,5,20]] Notably, this promoting effect was not observed for DOX@MSCPs and this effect was specific as expression of COX‐1, an isoenzyme of COX‐2, remained largely unchanged across the different treatments (Figure S4A, Supporting Information). These results indicate that celecoxib derivative cleaved from MSCPs effectively suppresses DOX‐induced COX‐2 upregulation and PGE_2_ production. The molecular mechanisms underlying this suppressive effect of celecoxib derivative are currently unclear and further study might be needed.

### 2.3 MSCPs Enhance DOX's Antitumor Activity via Celecoxib Inhibiting COX‐2/PGE_2_

**Figure 3:**
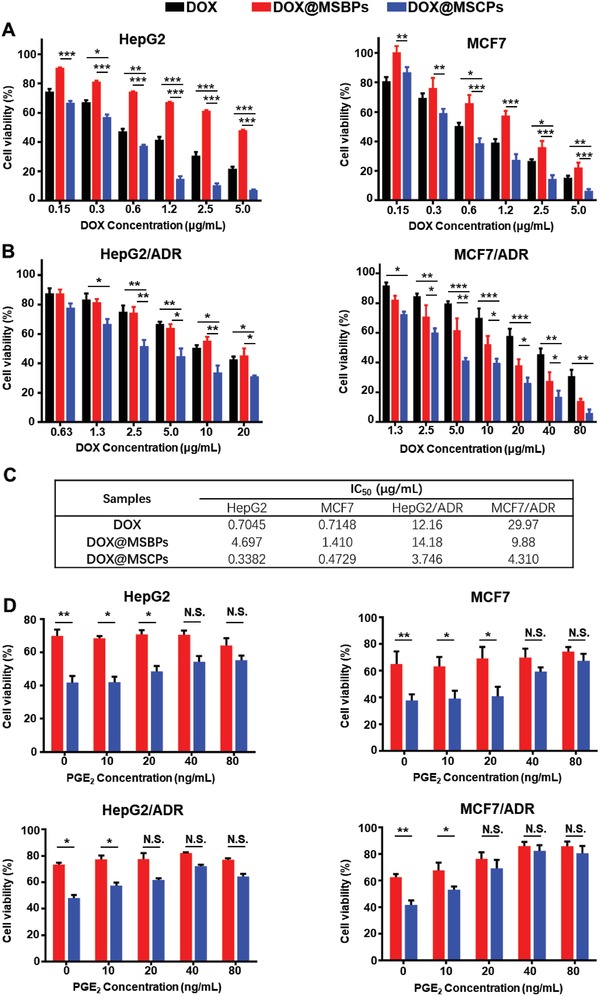
The enhanced antitumor activity of DOX@MSCPs. A) Cytotoxicity of DOX and DOX‐loaded nanoparticles in HepG2 cells and MCF7 cells after incubation for 48 h. B) Cytotoxicity of DOX and DOX‐loaded nanoparticles in HepG2/ADR and MCF7/ADR cells after incubation for 48 h. C) IC_50_ values (µg mL^−1^) of DOX, DOX@MSBPs and DOX@MSCPs in different cancer cell lines. D) Cytotoxicity of DOX@MSCPs in the presence of PGE_2_ (0–80 ng mL^−1^) in HepG2 and MCF7 (0.6 µg mL^−1^ of DOX in drug‐sensitive cancer cells), HepG2/ADR and MCF7/ADR (5 µg mL^−1^ of DOX in drug‐resistant cancer cells). Data shown as mean ± SD, *n* = 3 per treatment, *p < 0.05, **p < 0.01, ***p < 0.001, N.S., not significant.

DOX@MSCPs' antitumor activity was assessed in DOX‐sensitive cancer cells (HepG2 and MCF7 (breast cancer cell line)) and DOX‐resistant cancer cells (HepG2/ADR and MCF7/ADR). In DOX‐sensitive cancer cells, DOX@MSCPs showed significantly higher antitumor activity than free DOX and DOX@MSBPs (**Figure** 3A,C). Importantly, in DOX‐resistant cancer cells, DOX@MSCPs also exhibited potent therapeutic activity with IC_50_ approximately three‐ to seven‐fold lower than free DOX and two‐ to four‐fold lower than DOX@MSBPs (Figure 3B,C). Since MSCPs and MSBPs on their own did not kill cancer cells (Figure S5A, Supporting Information) and they exhibited the similar efficacy in cellular internalization and nuclear drug accumulation (Figure 2C–E), the enhanced therapeutic activity observed for DOX@MSCPs was attributable to celecoxib moieties that mediated COX‐2 inhibition. Indeed, this enhanced killing activity was associated with reduced PGE_2_ synthesis as evidenced by the fact that addition of exogenous PGE_2_ to these DOX‐based treatments decreased the antitumor activity of DOX@MSCPs in a dose‐dependent manner (Figure 3D; Figure S5B, Supporting Information), but did not affect the cytotoxicity of free DOX and DOX@MSBPs. Together, these results demonstrate that MSCPs significantly enhance DOX's antitumor activity through celecoxib inhibiting COX‐2 and subsequent PGE_2_ production.

### 2.4 MSCPs Suppress Cancer Stemness, Invasiveness, and Acquisition of Chemoresistance Induced by DOX‐Based Multiround Chemotherapy In Vitro

**Figure 4:**
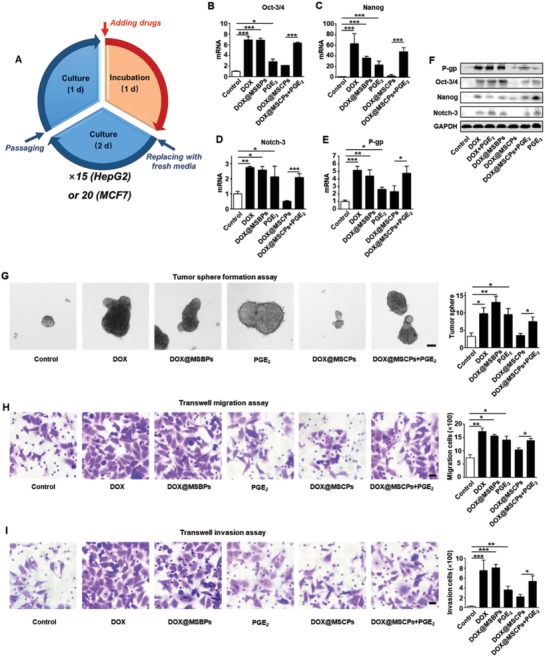
MSCPs suppressed cancer stemness and drug resistance induced by DOX‐based chemotherapy in vitro. A) Chemotherapy treatments for HepG2 cells (15 rounds) and MCF7 cells (20) in vitro. The cancer cells were exposed to chemotherapeutic formulations for 1 d and then incubated in fresh media for 2 d in each round. After 15 or 20 rounds, the cells were cultured in regular media for 1 week and then characterized. The relative mRNA levels of B) Oct‐3/4, C) Nanog, D) Notch‐3, and E) P‐gp in HepG2 cells after chemotherapy treatments (*n* = 3). F) The relative protein levels of P‐gp, Oct‐3/4, Nanog, and Notch‐3 in HepG2 cells after chemotherapy treatments. G) The tumor sphere formation of treated HepG2 cells over 10 d. The representative images and quantification of the resultant tumor spheres (*n* = 4). Scale bar, 50 µm. H) The migration ability of treated HepG2 cells. The representative images and quantification of migration cells (*n* = 4). Scale bar, 20 µm. I) The invasion ability of treated HepG2 cells. The representative images and quantification of invasion cells (*n* = 4). Scale bar, 20 µm. Data shown as Mean ± SD, *p < 0.05, **p < 0.01, ***p < 0.001.

To assess the impact of DOX@MSCPs on cancer stemness, metastasis, and drug resistance induced by chemotherapy, two types of cancer cells (HepG2 and MCF7) were treated with multiple rounds of chemotherapy that resembled clinical chemotherapy strategy (**Figure** 4A). Expression of Oct‐3/4, Nanog, and Notch‐3, three widely used core marker genes reflecting stemness, was assessed. Tumor‐sphere formation assay, a standard method for in vitro examination of self‐renewal and differentiation capabilities of cancer cells, was also performed. Free DOX and DOX@MSBPs significantly increased mRNA and protein levels of Oct‐3/4, Nanog, and Notch‐3 in HepG2 (Figure 4B–D,F) and MCF7 cells (Figure S6A–C,E, Supporting Information), and improved the efficacy of tumor‐sphere formation in both cell lines (Figure 4G; Figure S6F, Supporting Information), indicating that DOX‐based chemotherapy regimen enhances cancer stemness. In stark contrast, this enhancement was not observed for DOX@MSCPs treatment, suggesting that celecoxib moieties suppress DOX‐mediated stemness enhancement, presumably through COX‐2 inhibition. Supporting this notion, the addition of exogenous PGE_2_ into DOX@MSCPs treatment boosted the expression of cancer stemness core genes and increased the efficacy of tumor‐sphere formation to a level similar to DOX‐ or DOX@MSBPs‐treated cells. These results reveal that DOX@MSCPs abrogate DOX‐mediated enhancement on cancer stemness by suppressing PGE_2_ production via celecoxib.

Metastasizing to distant sites is a key pathological process leading to cancer relapse even after primary tumors are resected. We next used Transwell chamber assay with or without Matrigel to examine cancer cells' capabilities to migrate and invade through extracellular matrix. While DOX and DOX@MSBPs treatments significantly promoted the migration and invasion of HepG2 and MCF7 cells, MSCPs effectively inhibited this DOX‐conferred enhancement on migration and invasion in both cell lines (Figure 4H,I; Figure S7, Supporting Information). Such inhibition was abolished by addition of PGE_2_. Together, these observations demonstrate that celecoxib from DOX@MSCPs effectively curbs DOX‐induced enhancement on invasiveness.

P‐glycoprotein (P‐gp), a prominent transmembrane efflux pump mediating cancer multidrug resistance by pumping drugs out of cells, can be upregulated by chemodrugs through COX‐2/PGE_2_‐dependent mechanisms.[[qv: 10b,c,21]] Given MSCPs sensitized cancer cells to DOX through celecoxib inhibiting COX‐2/PGE_2_ (Figure 3), we tested whether this was due to P‐gp downregulation resulting from COX‐2 inhibition. In support of this notion, DOX@MSCPs treatment did not significantly upregulated P‐gp expression at both mRNA and protein levels (Figure 4E,F), whereas free‐DOX and DOX@MSBPs treatments drastically elevated P‐gp expression in HepG2 cells (Figure 4E,F) and in MCF7 cells (Figure S6D,E, Supporting Information). Importantly, P‐gp expression in DOX@MSCPs treated cells could be elevated by adding exogenous PGE_2_ (Figure 4E,F; Figure S6D,E, Supporting Information), consistent with the notion that COX‐2/PGE_2_ promotes P‐gp expression.^10^, ^22^ These results indicate that MSCPs enhance DOX's cancer‐cell killing activity because released celecoxib suppresses P‐gp upregulation induced by DOX‐based chemotherapy. Taken together, DOX@MSCPs effectively abrogated the DOX‐induced enhancement on cancer stemness, invasiveness, and drug resistance acquisition.

### 2.5 MSCPs are In Vivo Biocompatible and Effectively Deliver Celecoxib and Doxorubicin into Tumor Local

**Figure 5:**
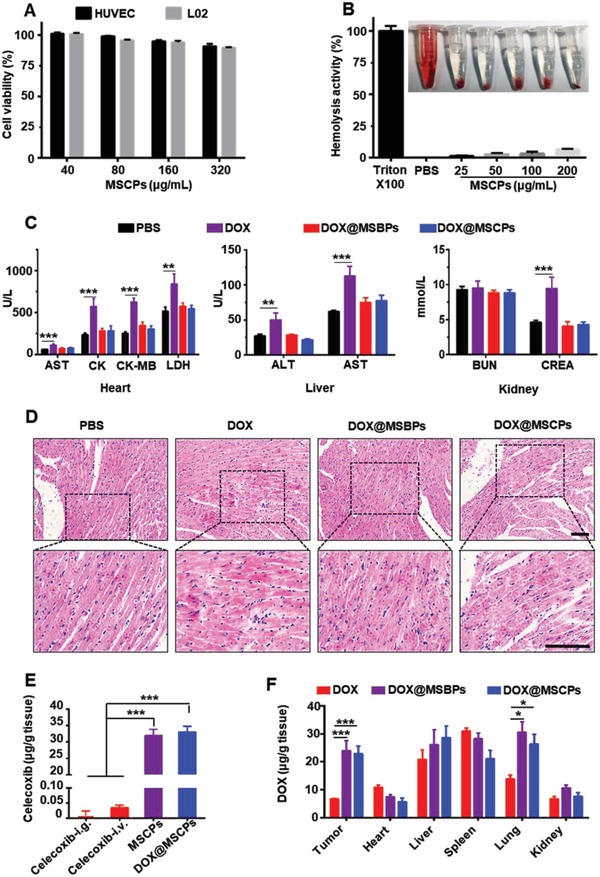
Biocompatibility of MSCPs and in vivo biodistribution of DOX@MSCPs. A) The cytotoxicity of MSCPs in HUVEC and L02 cells. B) Hemolysis assay of MSCPs. C) The biochemical analysis of the sera from the mice receiving treatment. Mice were treated with PBS, DOX, DOX@MSBPs or DOX@MSCPs for four times, and blood were collected at Day 21. Aminotransferase (ALT), aspartate aminotransferase (AST), creatine kinase (CK), creatine kinase‐MB (CK‐MB) and lactic dehydrogenase (LDH) was measured using U L^−1^, while creatinine (CREA) and blood urea nitrogen (BUN) was measured using mmol L^−1^. D) Hematoxylin and Eosin (HE) staining images of the cardiac tissues isolated from the mice receiving treatment. Scale bar, 50 µm. E) Celecoxib accumulation in tumor tissues of 4T1 breast cancer‐bearing mice after intragastric administration or intravenous injection of celecoxib, MSCPs or DOX@MSCPs (*n* = 3 per group; 5 mg kg^−1^ of celecoxib). F) Biodistribution of DOX in 4T1 breast cancer‐bearing mice receiving DOX‐based formulations (*n* = 3 per treatment). Data shown as mean ± SD, *n* = 3 per treatment, *p < 0.05, **p < 0.01, ***p < 0.001.

To study the biocompatibility of MSCPs, we first determined the cytotoxicity in human vascular endothelial cells (HUVEC cell line) and immortalized benign liver cells (L02 cell line). The viability of HUVEC and L02 cells was all higher than 85% after exposure to MSCPs for 48 h at the tested concentrations (40–320 µg mL^−1^) (**Figure** 5A), indicating a good cytocompatibility. Furthermore, we evaluated the hemolysis activity of MSCPs and found that there was no significant difference between the MSCPs and PBS treatments (Figure 5B), suggesting a good hemocompatibility.

We next assessed DOX@MSCPs' systemic toxicity. While free DOX significantly damaged the major organs, including heart, liver and kidney, DOX@MSCPs did not exhibit significant toxicity to these organs (Figure 5C). Moreover, while free DOX caused disorganization and swelling of myocytes in heart tissues, DOX@MSCPs well preserved myocardial structures (Figure 5D). DOX@MSCPs also did not cause weight loss in treated mice in the antitumor activity experiments (Figures S8C, S9C,D, and S10A, Supporting Information). These results indicate that MSCPs are biocompatible and effectively minimize systemic toxicity of carried chemotherapeutic agents.

To study whether DOX@MSCPs could effectively co‐deliver celecoxib and DOX to tumor sites in vivo, the biodistribution assays were performed in an orthotopic murine breast cancer model. Due to celecoxib's poor bioavailability and rapid clearance,^23^ drastically low levels of celecoxib (<0.01 µg g^−1^ by i.g. and < 0.04 µg g^−1^ by i.v.) were detected at tumor sites in the mice receiving celecoxib by intragastric administration and intravenous injection. In contrast, high concentrations of celecoxib (>30 µg g^−1^) were found within tumor mass in the mice receiving MSCPs or DOX@MSCPs (Figure 5E), likely because of enhanced permeability and retention effects of nanoparticles. Next, we evaluated DOX's biodistribution in tumor‐bearing mice. Compared to free DOX, the nanoparticles transported 3‐time more of DOX to tumor mass (Figure 5F). Further, more DOX was observed accumulating in lungs of the mice receiving DOX@MSCPs, suggesting that MSNs‐based delivery systems might be promising for treating lung metastasis. Together, these results indicate that DOX@MSCPs can effectively transport celecoxib and DOX to tumor local in vivo.

Then, we examined the in vivo antitumor effect of free DOX and free celecoxib. This combination exhibited the similar therapeutic activity as did free DOX alone (Figure S8A,B, Supporting Information), consistent with previous study.^23^ This result indicates that free celecoxib cannot enhance free DOX to suppress tumor growth, likely due to low accumulation of free DOX and free celecoxib in tumor local (Figure 5E,F). Thus, we did not include this treatment formulation in the following in vivo experiments in this study.

### 2.6 MSCPs Enhance In Vivo Antitumor Activity of DOX‐based Chemotherapy by Impairing DOX‐promoted Cancer Stemness, Metastasis, and Drug Resistance In Vivo

**Figure 6:**
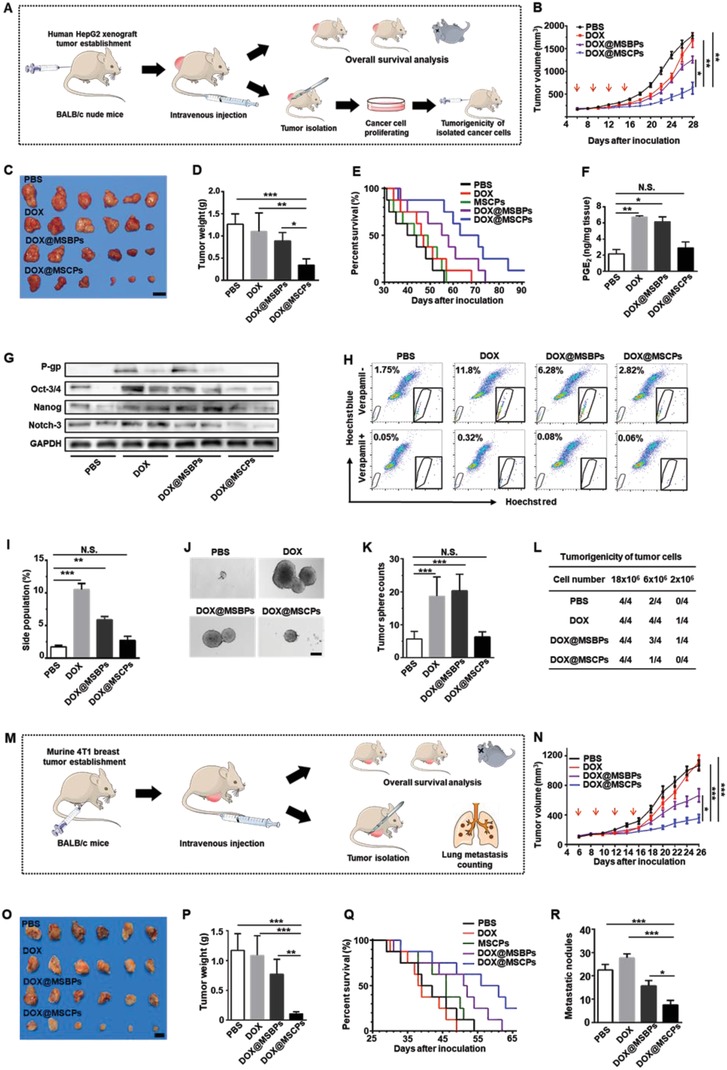
The antitumor activity of DOX@MSCPs in HepG2 tumor‐bearing BALB/c‐nude mice and 4T1 tumor‐bearing BALB/c mice. A) Schematic illustration of HepG2 xenograft tumor establishment, chemotherapy treatments, and subsequent analysis. B) Tumor growth of HepG2 tumor‐bearing mice treated with PBS, DOX, DOX@MSBPs, or DOX@MSCPs over 28 d (*n* = 8 per group). Red arrows indicate the time points for treatment (2 d apart, 4 times consecutively). C) Representative tumors isolated from the euthanized mice receiving treatment. Scale bar, 2 cm. D) Weights of the isolated tumors. E) Survival percentages of tumor‐bearing mice receiving treatment (*n* = 8 per group). F) PGE_2_ concentrations in tumor tissues of the mice receiving treatment (*n* = 3 per group). G) Protein levels of P‐gp, Oct‐3/4, Nanog, and Notch‐3 in tumor tissues of the mice receiving treatment. The tumors and cells were isolated at Day 18. H,I) Side population analysis of the isolated cancer cells from tumor tissues. J,K) The tumor sphere formation of the isolated cancer cells from tumor tissues. The isolated cancer cells were cultured in ultralow attachment dishes for 14 d, and the resultant spheres with a diameter over 50 µm were quantified (*n* = 3 per group). Scale bar, 150 µm. L) Tumorigenicity of the isolated cancer cells from tumor‐bearing mice receiving treatment. The isolated tumor cells were injected subcutaneously into BALB/c‐nude mice at the armpit of forelimb (*n* = 4 per group) and the formation of new tumors was examined at Day 14 after inoculation. M) Schematic illustration of 4T1 orthotopic breast tumor establishment, chemotherapy treatments, and subsequent analysis. N) Tumor growth in 4T1 tumor‐bearing mice treated with PBS, DOX, DOX@MSBPs or DOX@MSCPs over 26 d (*n* = 8 per group). Red arrows indicate the time points for treatment (2 d apart, 4 times consecutively). O) Representative tumors isolated from the euthanized mice receiving treatment. Scale bar, 1 cm. P) Weights of the isolated tumors. Q) Survival percentages of tumor‐bearing mice receiving treatments (*n* = 8 per group). R) Quantification of pulmonary metastatic nodules (*n* = 3 per group). Data shown as Mean ± SD, *p < 0.05, **p < 0.01, ***p < 0.001. N.S., not significant.

To comprehensively investigate in vivo antitumor effect of DOX@MSCPs, we employed two preclinical animal cancer models: human liver tumor‐bearing nude mice established using HepG2 cells (**Figure** 6A), and murine orthotopic breast cancer model established using 4T1 murine cell line (Figure 6M) (see Materials and Methods for details). When the tumor reached 150 mm^3^, the mice received chemotherapy treatments. Compared to PBS control, DOX@MSCPs potently reduced HepG2 tumor growth by 73% (Figure 6B–D), significantly higher than DOX (13% reduction) and DOX@MSBPs (30% reduction). Importantly, DOX@MSCPs drastically improved the overall survival of liver tumor‐bearing mice, much longer than free DOX and DOX@MSBPs did (Figure 6E). Similarly, in the orthotopic breast tumor‐bearing mice, DOX@MSCPs effectively suppressed primary tumor growth by 91%, significantly more effective than DOX (7% reduction) and DOX@MSBPs (34% reduction) (Figure 6N–Q). Since MSCPs by themselves did not exhibit antitumor activity (Figure S9A,B, Supporting Information), these results indicate that MSCPs release celecoxib to enhance in vivo antitumor efficacy of simultaneously delivered DOX, consistent with in vitro observations for MSCPs‐mediated augmentation of DOX's antitumor activity.

We hypothesized that such enhanced antitumor activity was possibly associated with celecoxib's in vivo impacts on cancer stemness and P‐gp expression. Indeed, highly consistent with the in vitro results (Figures 2 and 4), DOX@MSCPs suppressed PGE_2_ production, and abolished upregulation of P‐gp expression and cancer stemness core genes expression within HepG2 tumor mass (Figure 6F,G), indicating no enhancement in drug resistance capacity and cancer stemness. Consistently, the cancer cells isolated from the DOX@MSCPs‐treated mice contained a much smaller number of side population cells (a type of cancer stem‐like cells highly expressing P‐gp^24^) and exhibited dampened tumor‐sphere formation capability (Figure 6H–K). Further, as metastasis is a key contributor of cancer relapse,^25^ we then examined DOX@MSCPs' impact on distant metastasis in the murine orthotopic breast cancer model (Figure 6M) where lungs are the most often metastatic site. Compared to PBS control, DOX@MSCPs dramatically reduced the number of metastatic foci in lung by 67%, nearly two‐fold more effective than DOX@MSBPs treatment (Figure 6R; Figure S10B,C, Supporting Information). Together, these animal‐level, cell‐biological, and biochemical data collectively demonstrate that in remaining cancer cells, MSCPs effectively suppress cancer stemness, distant dissemination, and drug resistance capacity, which would otherwise be enhanced by DOX‐based chemotherapy.

Furthermore, given the impaired cancer stemness, we assumed that the tumorigenicity, a key feature tightly associated with cancer stemness, would be reduced accordingly in remaining cancer cells. To test this, the isolated cancer cells from tumors in the mice receiving the above treatments were subcutaneously inoculated back again into other nude mice. Strikingly, the cancer cells isolated from the DOX@MSCPs‐treated tumors formed new tumor mass with much less efficacy (0% at cell density of 2 × 10^6^; 25% at 6 × 10^6^) than the cells from the tumors treated with free DOX (25% at cell density of 2 × 10^6^; 100% at 6 × 10^6^) or DOX@MSBPs (25% at cell density of 2 × 10^6^; 75% at 6 × 10^6^) (Figure 6L). These observations reveal the impaired tumorigenic capability of DOX@MSCPs‐treated cells, suggesting a reduced risk of tumor recurrence.

### 2.7 DOX@MSCPs Inhibit Cancer Repopulation by Abrogating Development of Acquired Chemoresistance During Clinical‐Resembling Multiround Chemotherapy In Vivo

**Figure 7:**
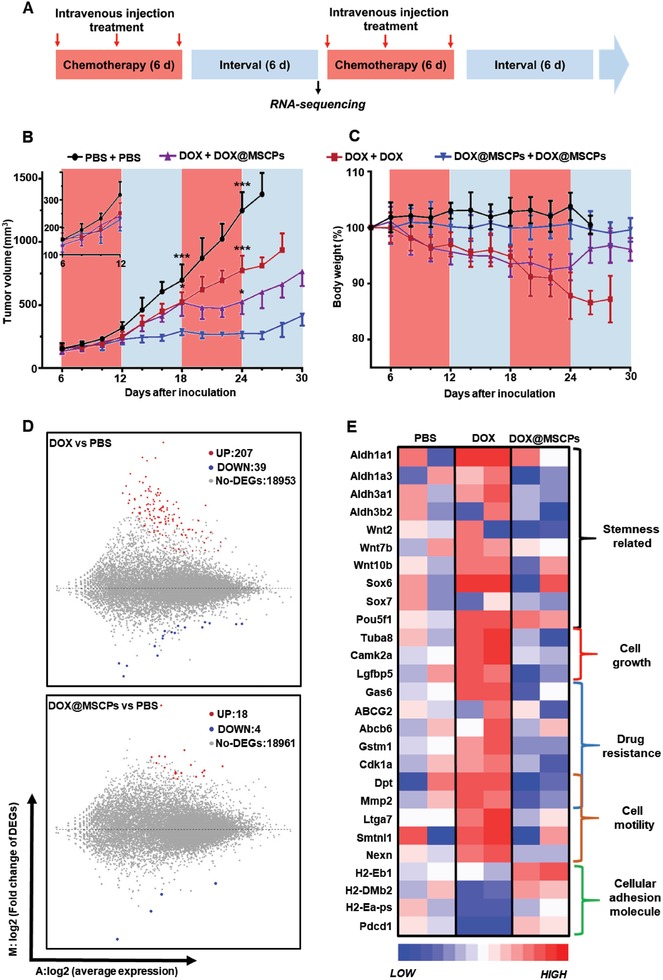
MSCPs suppressed the DOX‐based chemotherapy induced therapeutic resistance in murine breast tumor‐bearing mice. A) The experimental flowchart showing multiple rounds of chemotherapy regimens. B) Tumor growth in 4T1 murine breast cancer‐bearing mice receiving multiround treatments (*n* = 6 per group). C) Body weights of these tumor‐bearing mice. D) The fold changes of gene expression in tumor cells collected after the first round therapy at Day 18. The global genes expression was compared between DOX or DOX@MSCPs groups and PBS group. The significantly changed genes were sorted by log_2_‐Ratio ≥ 1 or ≤ −1. E) Heat map showing the upregulated and downregulated genes within the tumors from the mice receiving indicated treatments, which were laid out and classified with NCBI NR database by gene function. Data shown as mean ± SD. ***p < 0.001.

Although in the above experiments DOX@MSCPs‐treated cells exhibited reduced tumorigenic capacity, these cells were subject to only one round of chemo‐treatment, which was temporally and modally different from clinical chemotherapeutic regimen that is a relatively lengthy process consisting of multiple rounds of chemotherapy. During this process, tumor cells were intermittently under chemotherapeutic pressure and can develop acquired drug resistance that confers cancer cells with an adaptive capability of repopulating tumor mass even though in the middle of chemotherapy. To test whether DOX@MSCPs could effectively abrogate acquisition of drug resistance during multiround chemotherapy, we set up the murine orthotopic breast cancer model that was subject to multiround DOX‐based chemotherapy, resembling clinical chemotherapeutic regimen (**Figure** 7A). When tumor mass reached 150 mm^3^, the mice received a two‐round chemotherapy (Figure 7A). In the “free DOX + free DOX” regimen (one DOX round followed by another DOX round), the tumor was initially responsive to chemotherapy with a slow growth, but grew rapidly through the interval period, and evidently developed chemo‐resistance not responding well to DOX in the second DOX round (Figure 7B). However, when DOX@MSCPs replaced DOX in the second round (“DOX + DOX@MSCPs” regimen), the tumor growth was effectively curbed during the second chemotherapy round (Figure 7B), but resumed in the second interval, possibly because tumor stemness was insufficiently suppressed. Remarkably, in “DOX@MSCPs + DOX@MSCPs” regimen (Figure 7A), the tumor growth was significantly impaired throughout (Figure 7B). Meanwhile, DOX@MSCPs treatment did not result in weight loss that was observed in free‐DOX treated animals (Figure 7C). Together, these results indicate that DOX@MSCPs effectively debulk tumors, and suppress development of acquired chemo‐resistance, thus preventing tumor mass repopulation during and after treatment. Consistent with these observed phenotypical changes, global transcriptome profiling of the treated tumors revealed that DOX@MSCPs downregulated expression of the genes related to chemo‐resistance, cell growth, and cell motility without affecting expression of the cancer stemness core genes (Figure 7D,E), most of which were however largely upregulated in DOX‐treated tumors (Figure 7D,E; Figure S11–S14, Supporting Information). Moreover, DOX@MSCPs also enhanced expression of cell adhesion related genes that were dampened in DOX‐treated tumors (Figure 7D,E).

## 3 Conclusion

In summary, we offered a new approach to solve the limitation of conventional chemotherapy regimen that reduces tumor bulk while facilitates cancer relapse by enhancing stemness, metastatic capacity, and drug resistance in remaining cancer cells. By designing a smart drug delivery system with an ability of inhibiting COX‐2/PGE_2_ axis underpinning chemodrugs' “backdoor” effects, we successfully synthesized celecoxib‐conjugated mesoporous silica nanoparticles as a dual drug delivery system for celecoxib and DOX (MSCPs). MSCPs effectively encapsulated payloads, simultaneously released celecoxib derivative and DOX in response to intracellular redox environment, and effectively transported celecoxib and DOX to tumor local. Combining in vitro and in vivo three preclinical animal cancer models, we provided cell‐biological, biochemical, and genetic and molecular data that not only confirmed that conventional and nanomedicine‐based chemotherapy regimens promote cancer stemness and drug resistance to repopulate tumors, but also more importantly, demonstrate that MSCPs sufficiently abrogate DOX‐mediated enhancement on cancer stemness, metastasis and drug resistance through celecoxib derivative blocking the COX‐2/PGE_2_ pathway, which thus collectively improved the therapeutic efficacy of DOX‐based chemotherapy. Therefore, we developed a promising dual drug delivery platform for effectively reducing tumor burden while simultaneously reversing chemotherapy‐cultivated cancer stemness, metastasis and drug resistance.

## 4 Experimental Section

*Cell Culture and Animals*: Human breast cancer cells (MCF7 and MCF7/ADR cells) were cultured in Roswell Park Memorial Institute (RPMI) 1640 medium (10% fetal bovine serum (FBS), 100 unit mL^−1^ penicillin and 100 µg mL^−1^ streptomycin) at 37 °C. Human hepatocellular carcinoma cells (HepG2 and HepG2/ADR cells) were cultured in Dulbecco's Modified Eagle's Medium (DMEM) (10% FBS, 100 unit mL^−1^ penicillin and 100 µg mL^−1^ streptomycin) at 37 °C.

Athymic female BALB/c nude mice (5‐6 weeks) were purchased from Charles River Laboratories (Beijing, China). Female BALB/c mice (5 weeks) were purchased from Laboratory Animal Center, Huazhong University of Science and Technology (Wuhan, China). All mice were housed in specific pathogen free (SPF) condition in Laboratory Animal Center. All animal experiments were performed following the guidelines of the care and use of laboratory animals approved by the ethics committee of Tongji Medical College, Huazhong University of Science and Technology, Wuhan, China.

*In Vitro Drug Release*: To test the drug release behavior in vitro, DOX‐loaded nanoparticles (0.5 mg) were incubated in the buffers [PBS (pH 7.4) or acetate buffer (pH 5.0), 1.5 mL] with or without DTT (10 × 10^−3^ m), respectively. Next, these dispersions were shaken at 200 rpm, and the media containing released DOX were replaced with fresh buffers. The concentrations of DOX and celecoxib derivatives in the retractile media were examined using a UV–vis spectrophotometer (Perkin–Elmer Lambda Bio‐40 UV/Vis spectrometer) at 480 and 252 nm, respectively. The absorbance of DOX or celecoxib at 480 or 252 nm was recorded for establishing standard curves. Then, the drug concentration in each sample was calculated using the measured absorbance.

*Cellular Assay for COX‐2 Inhibiting Activity*: HepG2 cells were seeded in 6‐well plates and cultured for 24 h. Next, the cells were treated with PBS, DOX, DOX/CEL, DOX@MSCPs, DOX@MSBPs (0.5 µg mL^−1^ of DOX, 0.61 µg mL^−1^ of celecoxib) for 36 h. Then, the cell culture media were collected, and fresh media containing 100 × 10^−6^ m arachidonic acid were added and incubated with cells for 1 h. The PGE_2_ contents in these culture media were measured using ADI PGE_2_ Elisa kit (ADI, USA), and normalized to the number of live cells (determined by CCK8 assay). The COX‐1 and COX‐2 protein levels of treated cells were examined by Western blot analysis.

*Intracellular DOX Release*: HepG2 cells were seeded on the glass coverslips (10 mm^2^) in 12‐well plates (6 × 10^4^ cell per well) and cultured for 24 h. Next, the cell media were replaced with fresh DMEM containing free DOX, DOX@MSBPs, or DOX@MSCPs (with or without 1 × 10^−3^ m BSO) at DOX concentration of 4 µg mL^−1^. After incubation for 2 h, the cells were washed with PBS (3 times), fixed with 4% paraformaldehyde and stained with Hoechst 33 342 (10 µg mL^−1^). Finally, the fixed cells were imaged using Nikon Ti‐U microscope equipped with a CSU‐X1 spinning‐disk confocal unit (Yokogawa) and an EM‐CCD camera (iXon+; Andor). The fluorescence intensity of DOX in nucleus was quantified by ImageJ software.

*Cellular Uptake*: HepG2 cells were seeded in 6‐well plates (10^5^ cell per well) and cultured for 24 h. Next, the media were replaced with fresh DMEM containing free DOX, DOX@MSBPs, or DOX@MSCPs (with or without 1 × 10^−3^ m BSO) at DOX concentration of 4 µg mL^−1^. After incubation for 2 h, the cells were washed, harvested, and analyzed by flow cytometry (Canto II, BD Company, USA).

*In Vitro Cytotoxicity Assay*: The cytotoxicity of MSNs was accessed by MTT assay. Briefly, cells (HepG2, MCF7, HepG2/ADR, or MCF7/ADR) were seeded in 96‐well plates (8000 cells per well), respectively, and cultured for 24 h. Then, the cell media were replaced with fresh media containing DOX, DOX@MSBPs or DOX@MSCPs, respectively. After incubation for 48 h, the cells were washed and incubated in fresh media containing MTT (200 µL, 5 mg mL^−1^ of MTT) for another 4 h. Next, the media were carefully removed and replaced with DMSO (150 µL). After the produced formazan was dissolved, the absorbance at 490 nm was determined using a microplate reader (Infinite F50, Tecan, Switzerland).

To study the cytotoxicity of these DOX‐based formulations in the presence of PGE_2_, the cells were treated with DOX, DOX@MSBPs or DOX@MSCPs in the media containing exogenous PGE_2_ (10–80 ng mL^−1^). After incubation for 48 h, the relative viability of cells was examined by MTT assay as described above.

*In Vitro Multicycles Chemotherapy Assay*: To strictly simulate the clinical periodic chemotherapy regimen, the cancer cells (HepG2 and MCF7) were exposed to DOX‐based formulations with multicycles in vitro. Briefly, cells were seeded in 6‐well plates at a density of 10^5^ per well and cultured for 24 h. For each cycle treatment, the cells were treated with DOX, DOX@MSBPs, DOX@MSCPs, DOX@MSCPs plus PGE_2_, or PGE_2_ for 24 h (DOX, 0.2–0.4 µg mL^−1^. DOX concentration raised gradually during progress to kill portion of cancer cells and leave enough survival cells for passage and culture; PGE_2_, 40 ng mL^−1^), and subsequently incubated in fresh media for 48 h. Then the cells were passaged, seeded, and treated as described above for next cycle. HepG2 cells were treated with 15 cycles, and MCF7 cells were treated with 20 cycles in total. After these multicycle treatments, cells were characterized by q‐PCR, Western blot, tumor‐sphere formation, and transwell assays.

*Transwell Migration and Invasion Assay*: Cells migration and invasion assay were conducted on the Corning Transwell 3422 24‐well plates (8 µm pore size). The dispersed cells were seeded on the upper chamber in the media without FBS (10^5^ per well, 100 µL). The lower chamber was filled with fresh culture medium containing 10% FBS (500 µL). After incubation for 18 h, the cells were fixed with 4% paraformaldehyde and stained with 0.1% crystal violet staining solution. Cells stayed on the upper side of the membrane were wiped out with a cotton swab, and the cells migrated through the pores of polycarbonate membrane were imaged.

For the invasion assay, matrigel solution (50 µL, 11%, BD‐Matrigel) was added on the upside of polycarbonate membrane of upper chamber at 4 °C and gelled for 30 min at 37 °C. Then the cells were seeded as above procedure. The cells invaded through the matrigel and polycarbonate membrane were fixed, stained, imaged as described above.

*Tumor‐Sphere Formation Assay*: The tumor‐sphere formation assay was performed as our previous study.^26^ Briefly, the single suspended MCF7 (4000 cells per well) or HepG2 (9000 cells per well) cells were seeded in 24‐well ultralow attachment dishes (Corning, USA) and cultured in serum‐free X‐VIVO medium (Lonza, Switzerland) for 7 or 14 d. The spheres with diameter higher than 70 µm were imaged and counted.

*In Vivo Antitumor Activity Assay*: For HepG2 xenograft tumor model, HepG2 cells (1 × 10^7^ cells in 200 µL PBS) were subcutaneously injected in the right armpit of forelimb of 6‐week‐old female BALB/c nude mice. When the tumors reached to 150 mm^3^, mice were randomly divided into 5 groups (*n* = 11), and treated with PBS, DOX, MSCPs, DOX@MSBPs, or DOX@MSCPs (5 mg kg^−1^ of DOX or 6.1 mg kg^−1^ of celecoxib, two‐days apart, 4 times) through intravenous injection, respectively. Tumor sizes and body weights were measured every two days by a caliper, and the tumor volume was calculated by V = 0.5 × (width)^2^ × (length). At the 18th d after inoculation, three mice of each group were sacrificed randomly and the tumors were isolated for further characterizations. The protein and PGE_2_ levels of these tumor tissues were examined by western blot analysis and ELISA, respectively. To isolate the tumor cells, fresh tumor tissues were cut into pieces on ice, treated with collagenase, DNase and HAase at 37 °C for 1 h, then grinded and filtrated through a 70 µm cell strainer to obtain monodispersed cells. Finally, the cells were separated by Ficoll‐Paque Premium through gradient centrifugation method to obtain tumor cells in these tissues. The separated HepG2 cells were cultured in DMEM (20% FBS) for 3 d. Then, these cells were characterized by side population analysis, tumor‐sphere formation assay, and tumorigenicity assay. To assess the overall survival, tumor‐bearing mice were treated as described above (*n* = 8), and monitored.

For 4T1 breast metastasis tumor model, 4T1 cells (1.5 × 10^6^ in 150 µL PBS) were subcutaneously injected into the second right breast of female BALB/c mice. When the tumors reached to 150 mm^3^, mice were randomly divided into 4 groups (*n* = 8). The mice were treated with PBS, DOX, DOX@MSBPs, or DOX@MSCPs, respectively, as described above. At the 26th d after inoculation, the mice were sacrificed. Tumors and vital organs were isolated, weighted, imaged and fixed. Three lungs of each group were dissected, and metastatic nodules on the surface of lungs were isolated, counted and imaged. To assess the overall survival, tumor‐bearing mice were treated as described above (*n* = 8), and monitored.

For simulating clinical chemotherapy regimen, the 4T1 tumor bearing mice were received multicycles treatments. When tumor sizes reached to 150 mm^3^, tumor‐bearing mice were assigned into 4 groups randomly (*n* = 9), and treated with PBS (two cycles of PBS, 3 injections in each cycles), DOX (two cycles of free DOX, 3 injections in each cycles; 5 mg kg^−1^ of DOX), DOX‐DOX@MSCPs (one cycle of free DOX and one cycle of DOX@MSCPs, 3 injections in each cycles; 5 mg kg^−1^ of DOX), DOX@MSCPs (two cycles of DOX@MSCPs, 3 times i.v. injection each; 5 mg kg^−1^ of DOX). Each cycle of chemotherapy took 6 d, and separated by a 6‐day interval. At the 18^th^ d after inoculation, three mice of each groups were sacrificed randomly, and the tumor tissues were isolated and analyzed by RNA‐sequencing (BGI, China).

*Side Population Analysis*: Isolated tumor cells were dispersed in DMEM (2% FBS) and divided into two portions (with or without verapamil). Before stained with Hoechst 33 342, one portion of cells was pre‐treated with verapamil (100 × 10^−3^ m) for 10 min. Then, cells were treated with Hoechst 33 342 (5 µg mL^−1^, in DMEM) at 37 °C for 70 min, followed by washing with cold PBS (3 times) and stored at 4 °C. Next, the cells were stained by PI solution (0.2 µg mL^−1^) for 10 min and analyzed by flow cytometry.

*Tumorigenicity Assay*: The isolated HepG2 cells from tumor bearing mice were proliferated in vitro and subcutaneously injected (2–18 × 10^6^ cells) into the right infra‐axillary dermis of female BALB/c nude mice (*n* = 4). After two weeks, the tumor bearing mice (tumor volume > 100 mm^3^) were counted.

*RNA‐Sequencing*: Fresh tumors were isolated, cut into 100 mm^3^ tissues in ice, and washed by cold PBS. Then, these tumor tissues were frozen and transported in liquid nitrogen. The following quality testing, sequencing and genes expression analysis were performed in Beijing Genomics Institute (BGI, China). The data were laid out and classified using NCBI NR (nonredundant protein sequences from GenPept, Swissprot, PIR, PDF, PDB, and NCBI RefSeq; ftp.ncbi.nlm.nih.gov/blast/db/FASTA/).

*Biodistribution In Vivo*: 4T‐1 breast cancer model were constructed as above described. Once the tumor volume reached to 200 mm^3^, the mice were randomly divided into four groups (*n* = 3), and injected intravenously with celecoxib, MSCPs, DOX@MSCPs, or intragastrically administrated with celecoxib (5 mg kg^−1^ of celecoxib), respectively. After 24 h, the mice were sacrificed. Major organs and tumors were isolated, weighted, crushed with liquid nitrogen, re‐suspended in 20% NaOH solution for 12 h. The celecoxib in these tissues was then extracted using ethyl acetate and dried by nitrogen flow and vacuum. The residue was dissolved in methanol and analyzed by Waters 2695 Separation Module high‐performance liquid chromatography system using mixture of methanol/water (85/15) as the eluent (0.8 mL min^−1^; Waters C18 column, 4.6 × 250 mm), and detected with a photodiode array detector (Waters 2996) at 254 nm.

To assess the DOX biodistribution, 4T‐1 breast cancer model were generated. Once the tumor volume reached to 200 mm^3^, the mice were divided into 3 groups (*n* = 3) and injected intravenously with DOX, DOX@MSBPs, DOX@MSCPs (5 mg kg^−1^ of DOX), respectively. After 24 h, mice were sacrificed. The vital organs were separated, weighted, crushed with liquid nitrogen, and resuspended in PBS. The homogenates were separated by centrifugation (1200 rpm) and the supernatant was diluted with same volume of methanol. DOX concentrations in the supernatants were determined by fluorescent photometer (PerkinElmer Lambda 35; λ_ex/em_, 470/590 nm).

*Statistical Analysis*: Statistical analysis between groups was performed by two‐way ANOVA with a confidence interval of 95%. Data were shown as mean ± standard deviation (SD).

## Conflict of Interest

The authors declare no conflict of interest.

## Supporting information

Supplementary
