# Supplementary material for: Redox‐Responsive Dual Drug Delivery Nanosystem Suppresses Cancer Repopulation by Abrogating Doxorubicin‐Promoted Cancer Stemness, Metastasis, and Drug Resistance
Source: Adv Sci (Weinh). 2019 Feb 4;6(7):1801987. doi: 10.1002/advs.201801987 (PMC6446919; doi:10.1002/advs.201801987)
Supplement: Supplementary file 1 — Supplementary [file ADVS-6-1801987-s001.pdf]

Copyright WILEY-VCH Verlag GmbH & Co. KGaA, 69469 Weinheim, Germany, 2019.

ADVANCED SCIENCE Open Access

Supporting Information

for *Adv. Sci.,* DOI: 10.1002/advs.201801987

Redox-Responsive Dual Drug Delivery Nanosystem Suppresses Cancer Repopulation by Abrogating Doxorubicin-Promoted Cancer Stemness, Metastasis, and Drug Resistance

Jia Liu, Bingcheng Chang, Qilin Li, Luming Xu, Xingxin Liu, Guobin Wang, Zheng Wang,* and Lin Wang**

Supporting information

Redox-responsive dual drug delivery nanosystem suppresses cancer repopulation by abrogating doxorubicin-promoted cancer stemness, metastasis, and drug resistance

Jia Liu[#], Bingcheng Chang[#], Qilin Li, Luming Xu, Xingxin Liu, Guobin Wang*, Zheng Wang*, Lin Wang*

Dr. J. Liu, B. Chang, Dr. Q. Li, Dr. L. Xu, X. Liu, Prof. Z. Wang, Prof. L. Wang,
Research Center for Tissue Engineering and Regenerative Medicine, Union Hospital, Tongji Medical College, Huazhong University of Science and Technology, Wuhan, 430022, China;
Email: zhengwang@hust.edu.cn; lin_wang@hust.edu.cn;

Dr. Q. Li, Prof. L. Wang
Department of Clinical Laboratory, Union Hospital, Tongji Medical College, Huazhong University of Science and Technology, Wuhan, 430022, China.

Prof. G. Wang, Prof. Z. Wang
Department of Gastrointestinal Surgery, Union Hospital, Tongji Medical College, Huazhong University of Science and Technology, Wuhan, 430022, China; Email: wgb@hust.edu.cn

Materials: Hexadecyltrimethylammonium bromide (CTAB), tetraethylorthosilicate (TEOS), 3-merraptnpropylt rimethnxysilane (MPTMS), 2,2'-dithiodipyridine, 1-(3-dimethylaminopropyl)-3-ethylcarbodiimide (EDC/HCl), N-hydroxysuccinimide (NHS), and benzoyl chloride were purchased from Aladdin Chemical Co. (Shanghai, China). β-Cyclodextrin, epichlorohydrin, cysteamine hydrochloride, and celecoxib were purchased from Energy chemical (Shanghai, China). Doxorubicin was purchased from Meilun Biology Technology Co., Ltd (Dalian, China). PGE_2 and arachidonic acid were purchased from TCI chemicals (Shanghai, China). All the used solvents were analytical reagent and purchased from Sinopharm Chemical Regent (Shanghai, China). S-(2-aminoethylthio)-2-thiopyridine hydrochloride (SATH) was synthesized as the literature. Oct-3/4 (A7920), Notch-3 (A0929), and Nanog (A3232) were obtained from ABclonal Biotechnology Co. Ltd (USA). P-gp (ab170904) was obtained from Abcam Plc. (UK). COX-1 (CSB-PA164506) and COX-2 (CSB-MA000320) were obtained from CusAb (China).

Synthesis of MSNs-SH: The MCM-41 mesoporous silica nanoparticles with CTAB template (CTAB@MSNs) were synthesized as our previous work.[1] The synthesized CTAB@MSNs (0.5 g) and MPTMS (2.5 mL) were dispersed in methanol (100 mL), and stirred at room temperature overnight. Then CTAB@MSNs-SH were collected through washing and centrifugation. To remove the CTAB template, CTAB@MSNs-SH were dispersed in methanol (95 mL) containing hydrochloric acid (38%, 6 mL), and refluxed at 80 ^{o}C for 2 d. After washing with water and methanol, MSNs-SH were collected by centrifugation at 8000 rpm for 15 min, and dried by vacuum.

Synthesis of MSNs-SS-NH_2: The obtained MSNs-SH (0.32 g) were dispersed in methanol (40 mL), then SATH (0.32 g, 1.5 mmol) was added and the suspension was stirred at room temperature for 18 h. The nanoparticles were collected by centrifugation, washed with water and methanol, and finally dried by vacuum.

Synthesis of celecoxib succinamidic acid (CEL): Briefly, celecoxib (1.0 g, 2.6 mmol), succinic anhydride (1.3 g, 13 mmol) and triethylamine (0.5 mL) were dissolved in dichloromethane (50 mL), and stirred at room temperature for 24 h. After reaction, the solvent was removed under vacuum and the crude product was purified by column chromatography using the mixture of n-hexane and ethyl acetate (3:1) as eluent. The product was characterized by ^{1}H NMR (Figure S1).

Synthesis of MSNs-SS-CEL: Celecoxib succinamidic acid (0.2 g, 0.4 mmol), EDC (0.1 g, 0.5 mmol) and NHS (0.05 g, 0.4 mmol) were dissolved in anhydrous DMSO (15 mL), and stirred for 4 h at room temperature. Then, the mixture was added into MSNs-SS-NH_2 (0.2 g) suspension in anhydrous DMSO (10 mL), and stirred at room temperature for 24 h. The MSNs-SS-CEL were collected, washed, dried as above described.

Synthesis of MSNs-SS-Bz: MSNs-SS-NH_2 (0.2 g) were dispersed in anhydrous DMF (30 mL) with DIPEA (0.5 mL), then benzoyl chloride (200 μL in 5 mL DMF) was added dropwise to the dispersion in an ice bath and stirred at room temperature for 12 h. The MSNs-SS-Bz were obtained by washing and drying.

Doxorubicin loading and PCD wrapping: The silica nanoparticles (MSNs-SS-CEL and MSNs-SS-Bz, 200 mg) were dispersed in phosphate buffered saline (PBS, pH 7.4, 100 mL), respectively. Subsequently, doxorubicin solution (50 mg, in 10 mL water) was added and the suspension was stirred for 24 h. Then, poly(β-cyclodextrin) (PCD, 300 mg) was added and the suspension was stirred for another 24 h. Next, the DOX-loaded nanoparticles were washed (DOX@MSCPs and DOX@MSBPs) with PBS and water, and obtained by lyophilization. The PCD wrapped MSNs without DOX (MSCPs and MSBPs) were prepared with the same method without DOX loading procedure. The DOX loading contents of DOX@MSCPs and DOX@MSBPs were determined using a UV-Vis spectrophotometer (Perkin–Elmer Lambda Bio-40 UV/Vis spectrometer) at 480 nm.

Characterizations of MSNs: The zeta potential and particle size of nanoparticles were determined using Nano-ZS ZEN3600 (Malvern Instruments). Thermal gravimetric analysis (TGA) was conducted on METTLER TOLEDO TGA/DSC 1 actions. N_2 adsorption/desorption isotherm analysis were carried out using an adsorption analyzer (JW-BK112, Beijing JWGB Sci.&Tech. Co.,Ltd.). The morphology and cellular distribution of nanoparticles were observed using transmission electron microscope (TECNAI G2 20 TWIN). 2D ^{1}H nuclear Overhouser effect spectroscopy (NOESY) spectum was recorded using Bruker Avance III-600 MHz spectrometer (Bruker Biospin, Germany).

Quantitative real-time PCR: Total mRNA in cells or tumor tissues was extracted using Trizol reagent (Invitrogen, USA) and reversed into cDNA using Hiscript II reverse transcriptase (Vazyme Biotech, China). qPCR was performed on ABI StepOne Plus Detector System (ABI, USA) according to the instructions with AceQ qPCR SYBR® Green Master Mix (Vazyme Biotech, China). Relative expressions of genes were normalized using the house-keeping gene GAPDH as a reference. The primers were presented in **Table S1**.

Western blot analysis**:** Western blot protein analysis was carried out according to our previous study.[2] Briefly, cells or tissues were washed with PBS and lysed by RIPA Lysis Buffer (Beyotime, China; 50 μL, with protease inhibitor). After sonication in the ice bath, the supernatants containing proteins were acquired by centrifugation under 4 ^{o}C. Followed by protein measurement by BCA kit, and normalized to uniform concentration, the supernatants were mixed with loading buffer and boiled for 10 min. After electrophoresis in 10% SDS-PAGE gel, the proteins were transferred to NC membranes, followed by primary monoclonal antibody treatment (P-gp, Oct-3/4, Nanog, Notch-3, COX-1, COX-2, GAPDH), then incubated with horseradish peroxidase-coupled secondary antibody. The protein was measured followed by the BeyoECL Plus (Beyotime, China) reagent instructions.

Hemolysis assay: As the previous study,[3] the rabbit red blood cells (RBCs) suspension

(2% in PBS, v/v) was mixed with of MSCPs (100 μL in PBS), and shaken at 37 ^{o}C for 5 h. Next, the samples were centrifuged to separate the supernatants. The absorbance of supernatants at 545 nm was determined using a UV-Vis spectrophotometer (SHIMADZU UV-1800, Japan).

Biochemical analysis: The tumor-free female BALB/c mice were randomly divided into 4 groups, and treated with PBS, DOX, DOX@MSBPs, or DOX@MSCPs (5 mg/kg of DOX; 4 injections), respectively. At the 21th day after treating, blood samples were collected and underwent biochemical analysis (Beckman 583, USA), including aspartate aminotransferase (AST), creatine kinase (CK), creatine kinase isoenzymes (CK-MB), lactate dehydrogenase (LDH), alanine aminotransferase (ALT), aspartate aminotransferase (AST), blood urea nitrogen (BUN) and creatinine (CREA).

In vivo antitumor activity assay for combination of doxorubicin and celecoxib: The 4T1 cells (10^6 cells in 150 μL PBS) were subcutaneously injected into the second right breast of female BALB/c mice. When the tumors reached to 100 mm^3, mice were randomly divided into 4 groups and were treated with PBS (n=3), DOX (n=5), DOX plus celecoxib (n=5), and DOX@MSCPs (n=5) through intravenous injection, respectively (5 mg/kg of DOX or 6.1 mg/kg of celecoxib, two-days apart, 4 times). Tumor sizes and body weights were measured every two days by a caliper. At the 26th d after inoculation, the mice were sacrificed, and tumors were isolated and weighted.

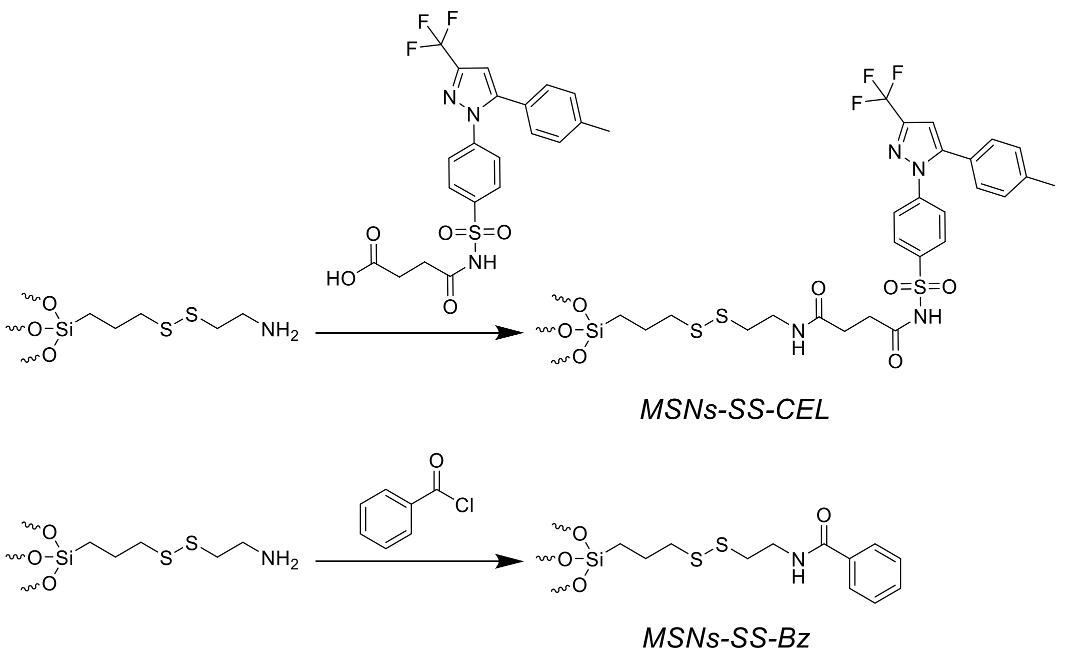

Scheme S1. The synthetic illustrations of MSNs-SS-CEL and MSNs-SS-Bz.

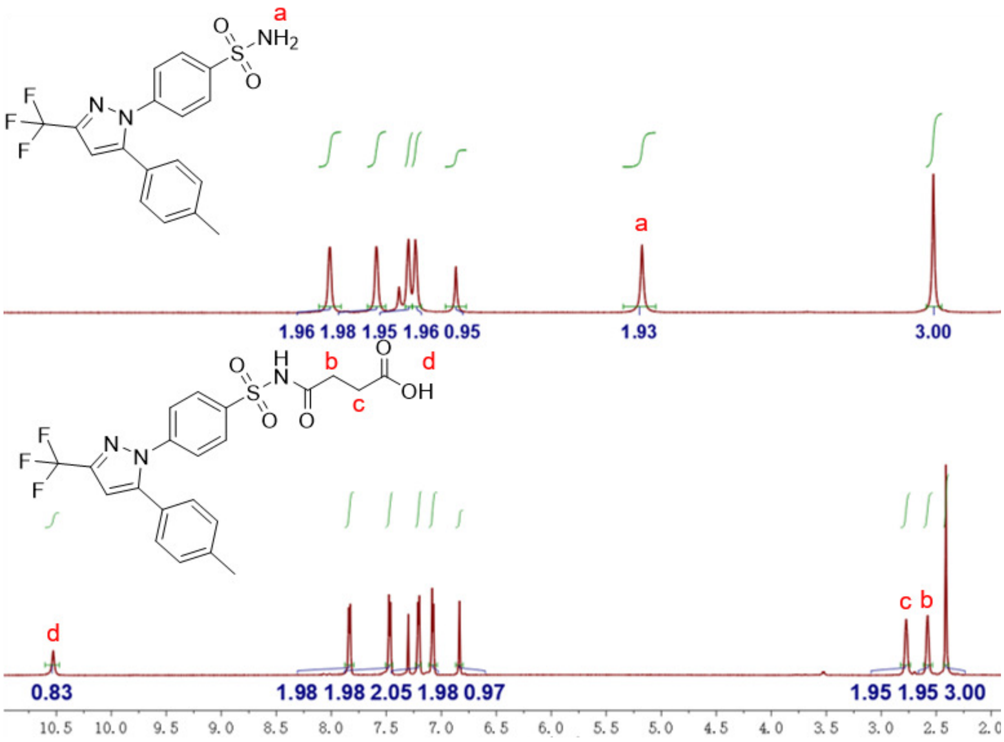

Figure S1. ^{1}H NMR spectra of celecoxib and celecoxib succinamidic acid (CEL) in $CDCl_3$.

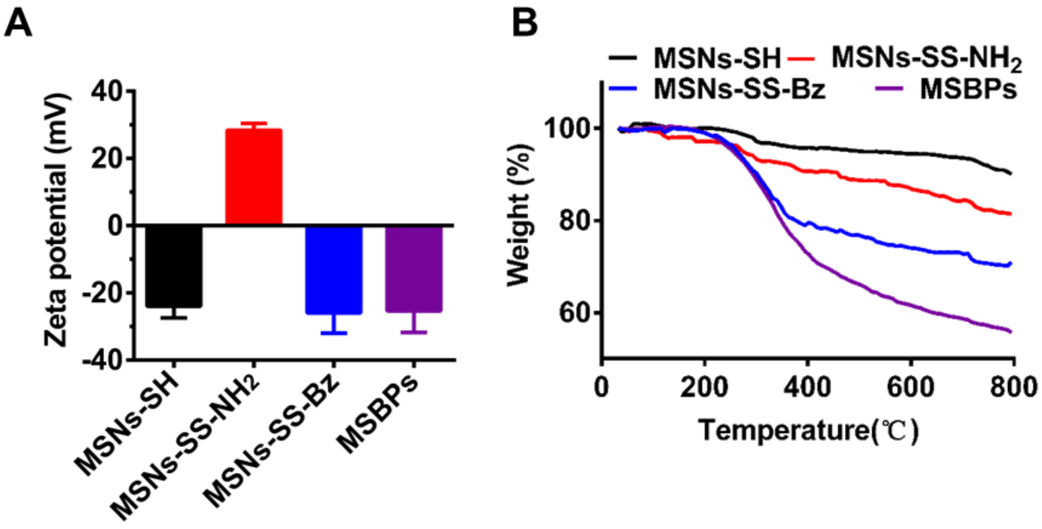

Figure S2. (A) Zeta potential of MSNs-SH, MSNs-SS-NH_2, MSNs-SS-Bz and MSBPs (n=3). Data shown as Mean ± SD. (B) TGA analysis of MSNs-SH, MSNs-SS-NH_2, MSNs-SS-Bz and MSBPs.

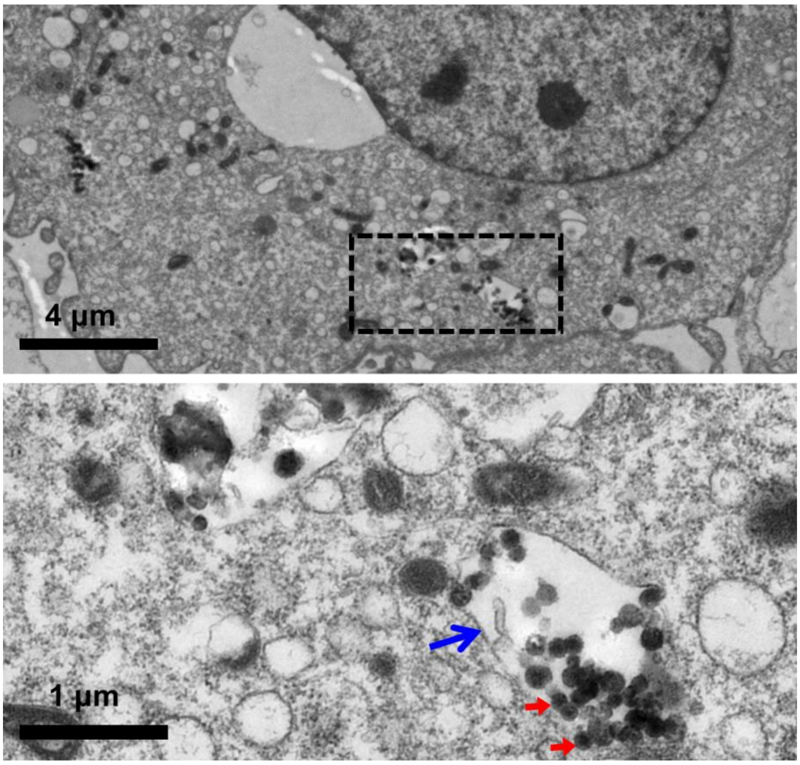

Figure S3. TEM images of HepG2 cells treated with DOX@MSCPs for 6 h. DOX@MSCPs were located in endosomes which were going to fuse with lysosomes. The black box outlined region is enlarged to the bottom, where the red arrows indicate the DOX@MSCPs in a endosome pointed by a blue arrow.

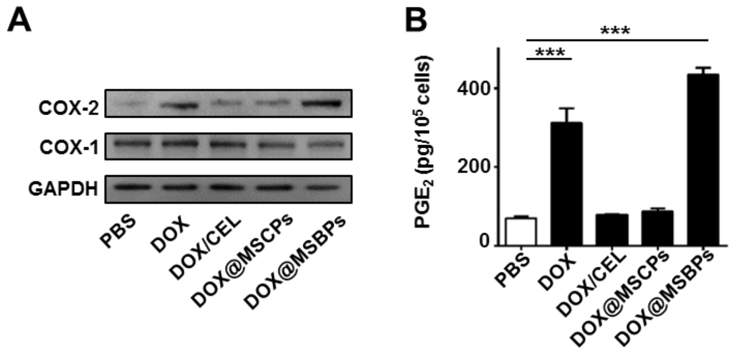

Figure S4. (A) COX-2 and COX-1 protein levels in HepG2 cells after being treated with DOX, DOX/ CEL (DOX and celecoxib succinamidic acid), or DOX-loaded nanoparticles for 36 h. (B) Quantification of PGE_2 released from HepG2 cells that were pretreated with DOX, DOX/CEL, or DOX-loaded nanoparticles for 36 h, and then incubated with fresh media containing arachidonic acid (100 μM) for 1 h. PGE_2 contents were normalized to live cell numbers and indicated as pg/10^5 cells (n=3). Data shown as Mean ± SD, ***p<0.001.

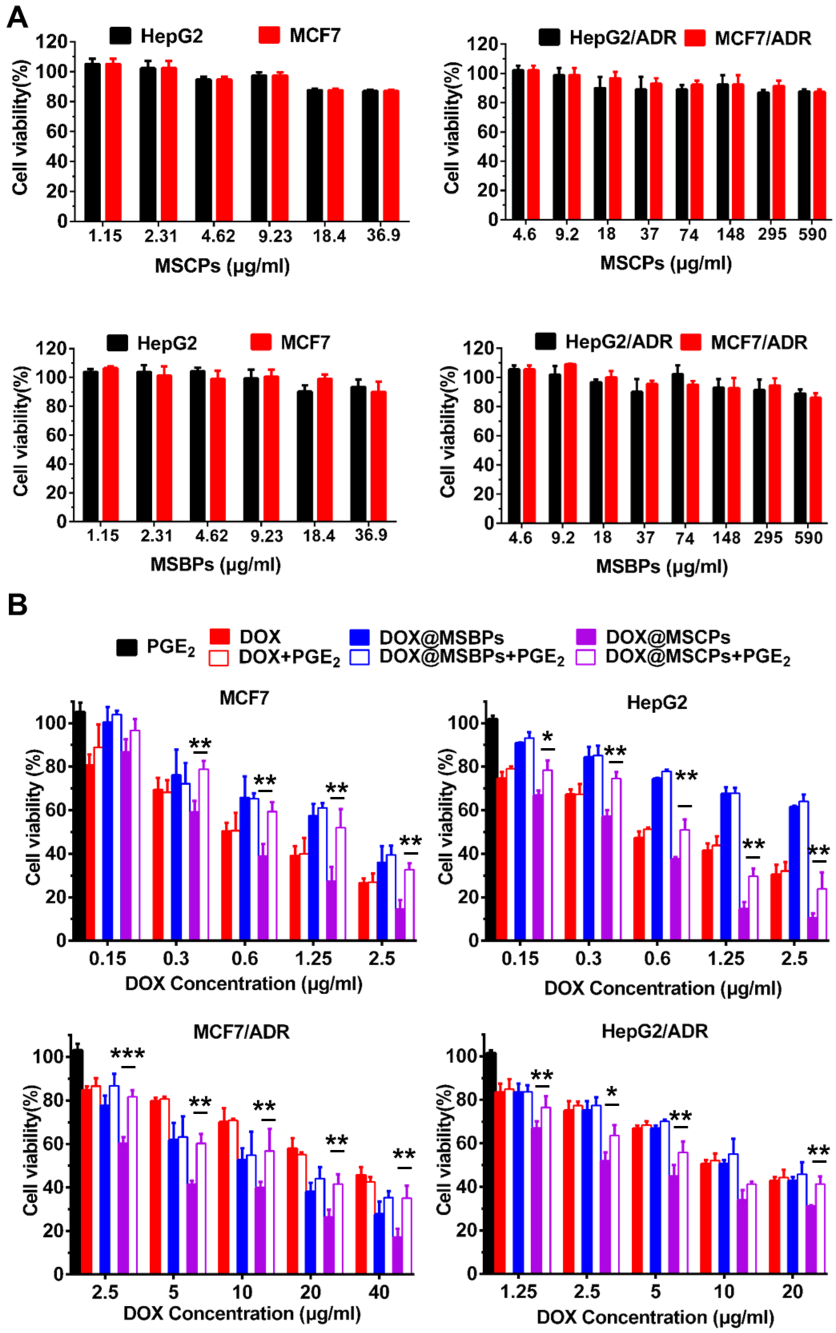

Figure S5. (A) The cytotoxicity of MSCPs and MSBPs in different cancer cell lines. (B) Cytotoxicity of DOX, DOX@MSBPs and DOX@MSCPs in the drug-sensitive (MCF7 and HepG2) and drug-resistant (MCF7/ADR and HepG2/ADR) cancer cells in the absence or presence of PGE_2 (20 ng/mL) after incubation for 48 h. Data shown as Mean ± SD, n=3 per treatment, *p<0.05, **p<0.01, ***p<0.001.

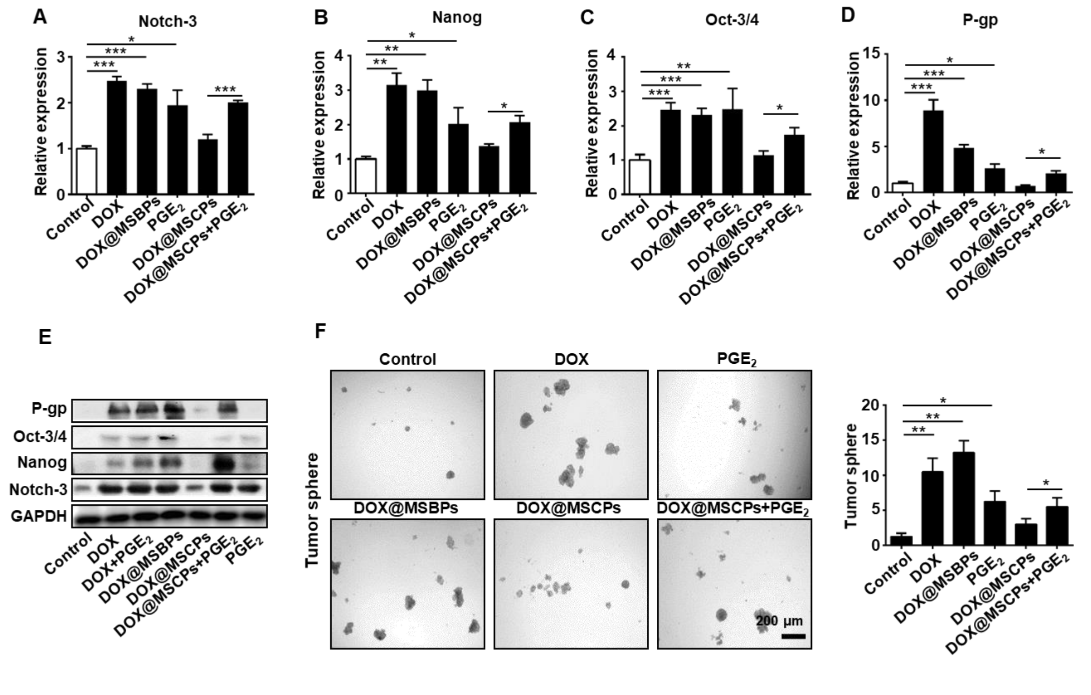

Figure S6. (A-D) The relative mRNA levels of Notch-3 (A), Nanog (B), Oct-3/4 (C), and P-gp (D) in MCF7 cells after chemotherapy treatments (n=3). (E) The relative protein levels of P-gp, Oct-3/4, Nanog and Notch-3 in MCF7 cells after chemotherapy treatments. (F) The tumor sphere formation of treated MCF7 cells over 10 d. The representative images and quantification of the resultant tumor spheres (n=4). Scale bar, 200 μm. Data shown as Mean ± SD, *p<0.05, **p<0.01, ***p<0.001.

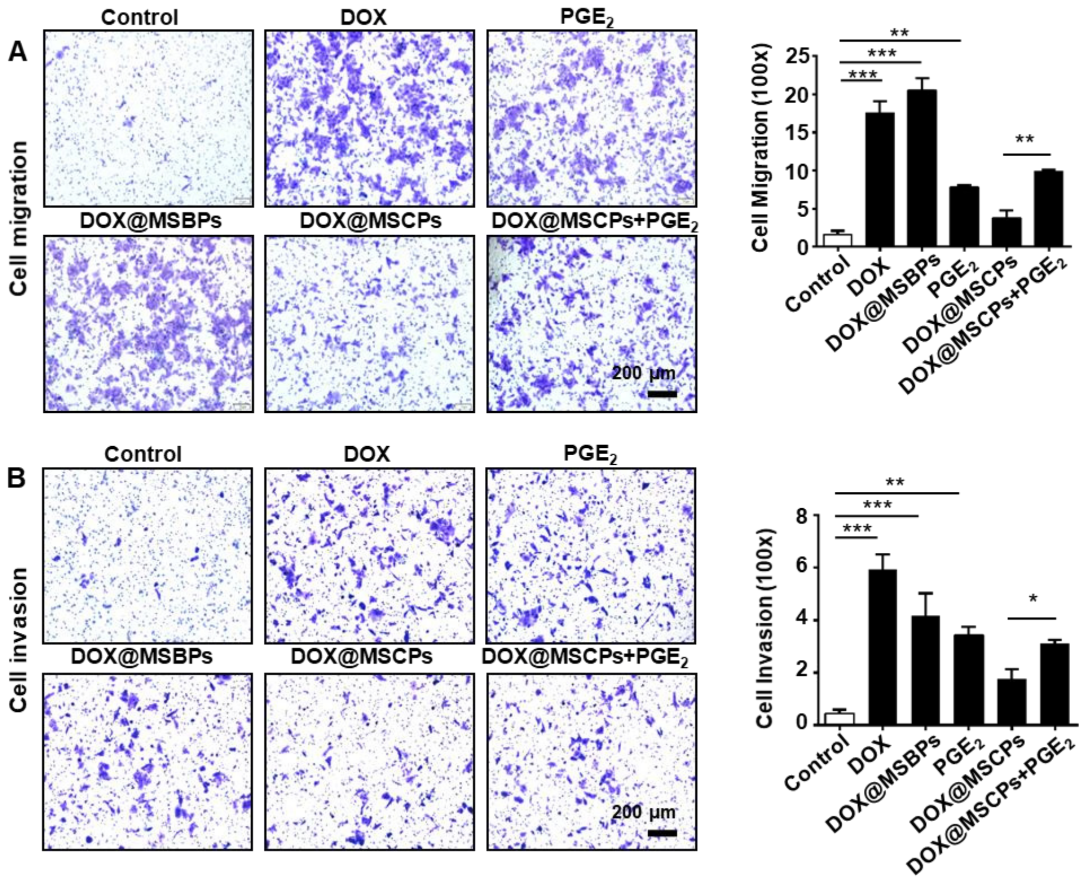

Figure S7. The migration (A) and invasion (B) ability of treated MCF7 cells. The representative images (left panel) and quantification (right panel) of the average number of migration/ invasion cells from 20 images. Scale bar, 200 μm. Data shown as Mean ± SD, n=4, *p<0.05, **p<0.01, ***p<0.001.

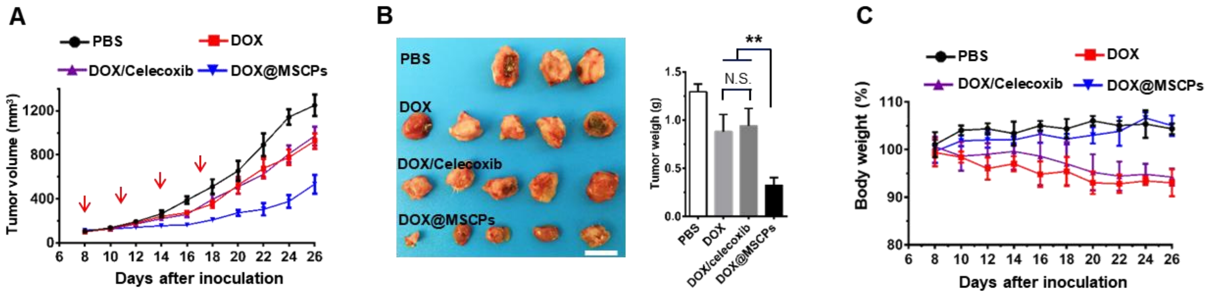

Figure S8. (A) Tumor growth of 4T1 tumors in the mice treated with PBS, DOX, DOX/Celecoxib or DOX@MSCPs over 24 d (n=3 for PBS; n=5 for DOX, DOX/Celecoxib and DOX@MSCPs, 5 mg/kg of DOX, 6.1 mg/kg of celecoxib). Red arrows indicate the time points for treatment. (B) Representative 4T1 tumors isolated from the euthanized mice in (A) (left panel). Scale bar, 1 cm. The weights of isolated tumors were measured (right panel). (C) Body weights of tumor-bearing mice treated with PBS, DOX, DOX/Celecoxib or DOX@MSCPs. Data shown as Mean ± SD, N.S., not significant, **$p<0.01$.

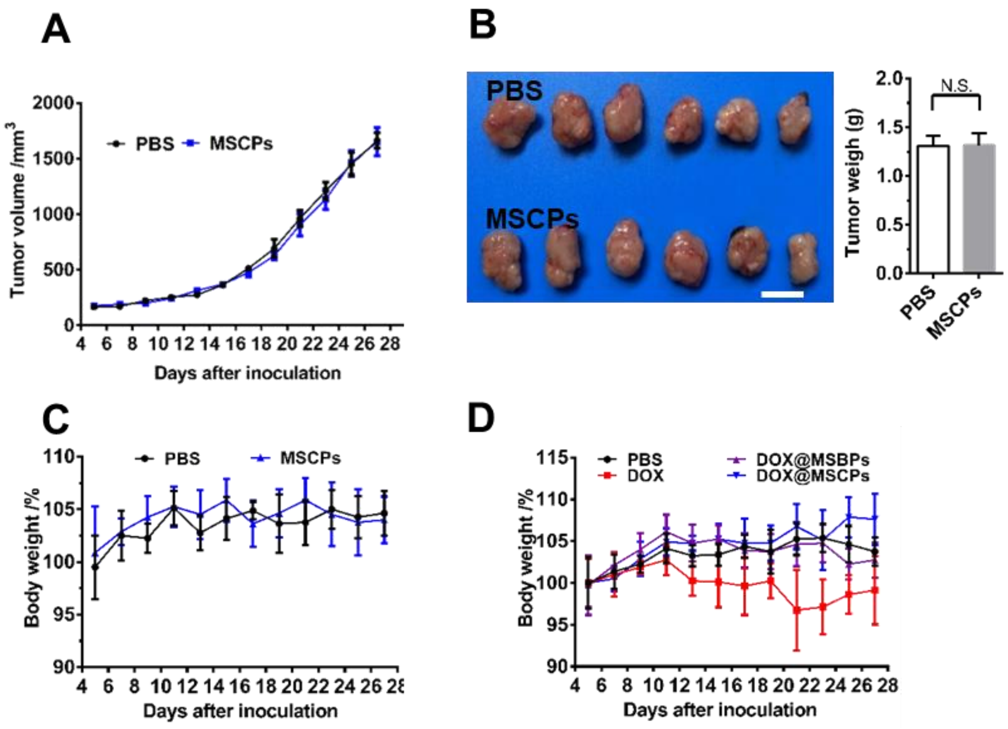

Figure S9. (A) Tumor growth of HepG2 tumor-bearing mice treated with PBS or MSCPs over 28 d (n=8). (B) Representative HepG2 tumors isolated from the euthanized mice receiving treatment (left panel). Scale bar, 8 mm. The weights of isolated tumors were measured (right panel). (C) Body weights of tumor-bearing mice treated with PBS or MSCPs. (D) Body weights of tumor-bearing mice treated with PBS, DOX, DOX@MSBPs or DOX@MSCPs. Data shown as Mean ± SD, N.S., not significant.

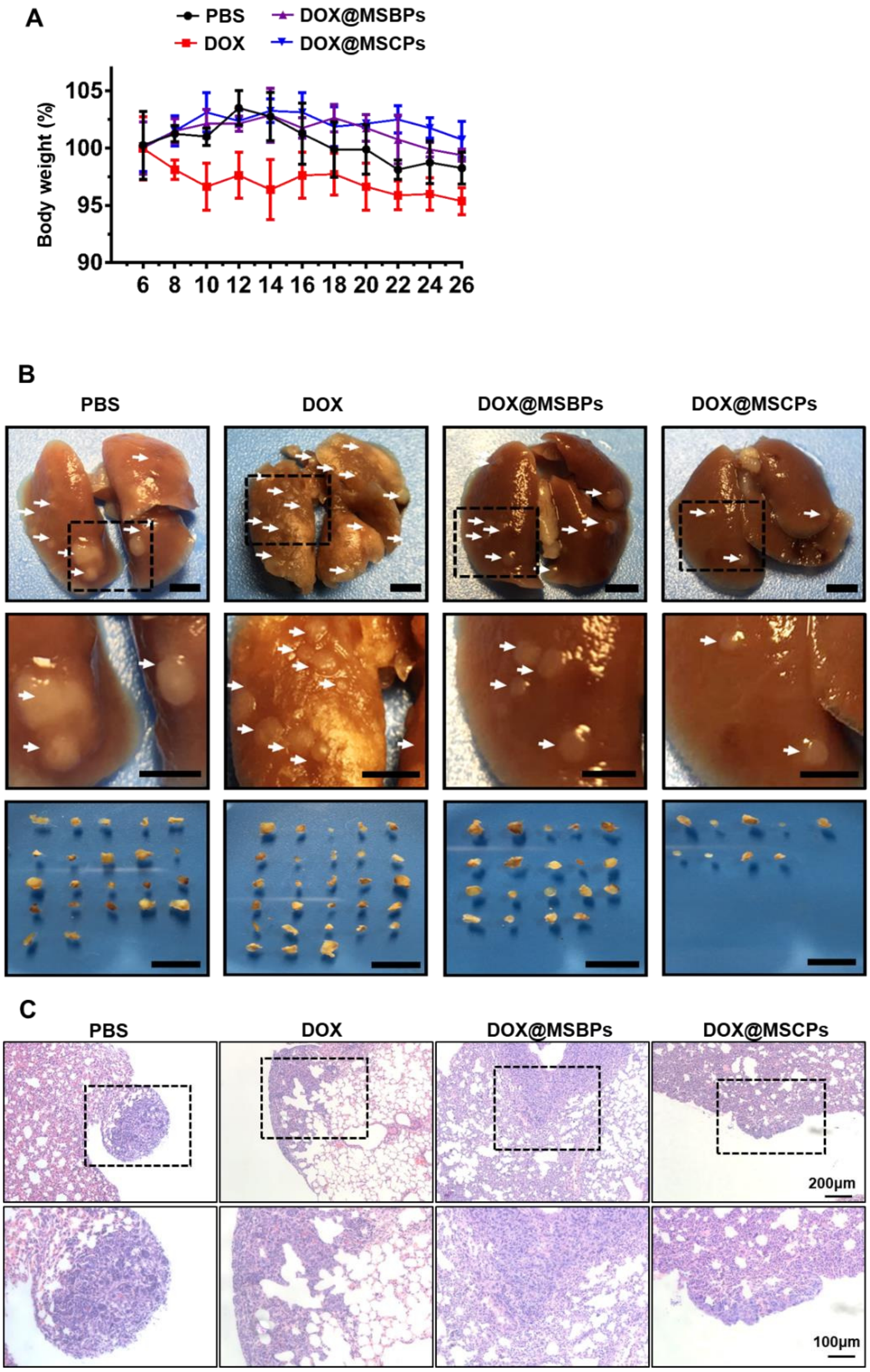

Figure S10. (A) Body weights of the 4T1 tumor-bearing mice treated with PBS, DOX, DOX@MSBPs or DOX@MSCPs. Data shown as Mean ± SD, n=8. (B) Representative images and quantification of pulmonary metastasis nodules. Scale bar, 1 mm. (C) Hematoxylin-Eosin staining images of the lung tissues in 4T1 tumor-bearing mice.

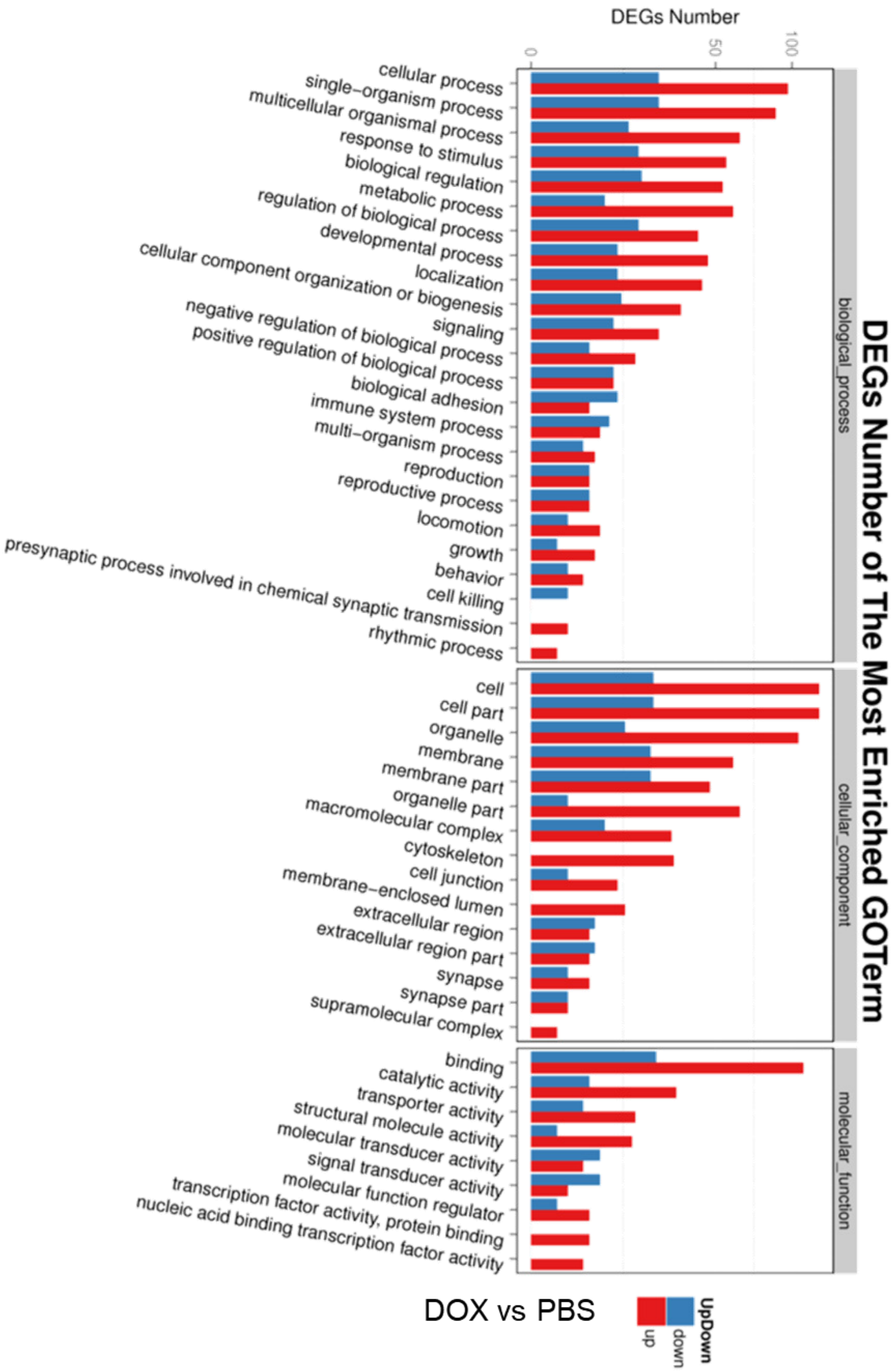

Figure S11. The globe gene changes between the mice receiving DOX and PBS treatments (n=2): Different expressed genes classified by biological functions, cellular component, and molecular functions based on GO annotation. Up: $\log_2$FoldChange≥1, Padj≤0.05; Down:

log$_2$FoldChange≤-1, Padj≤0.05.

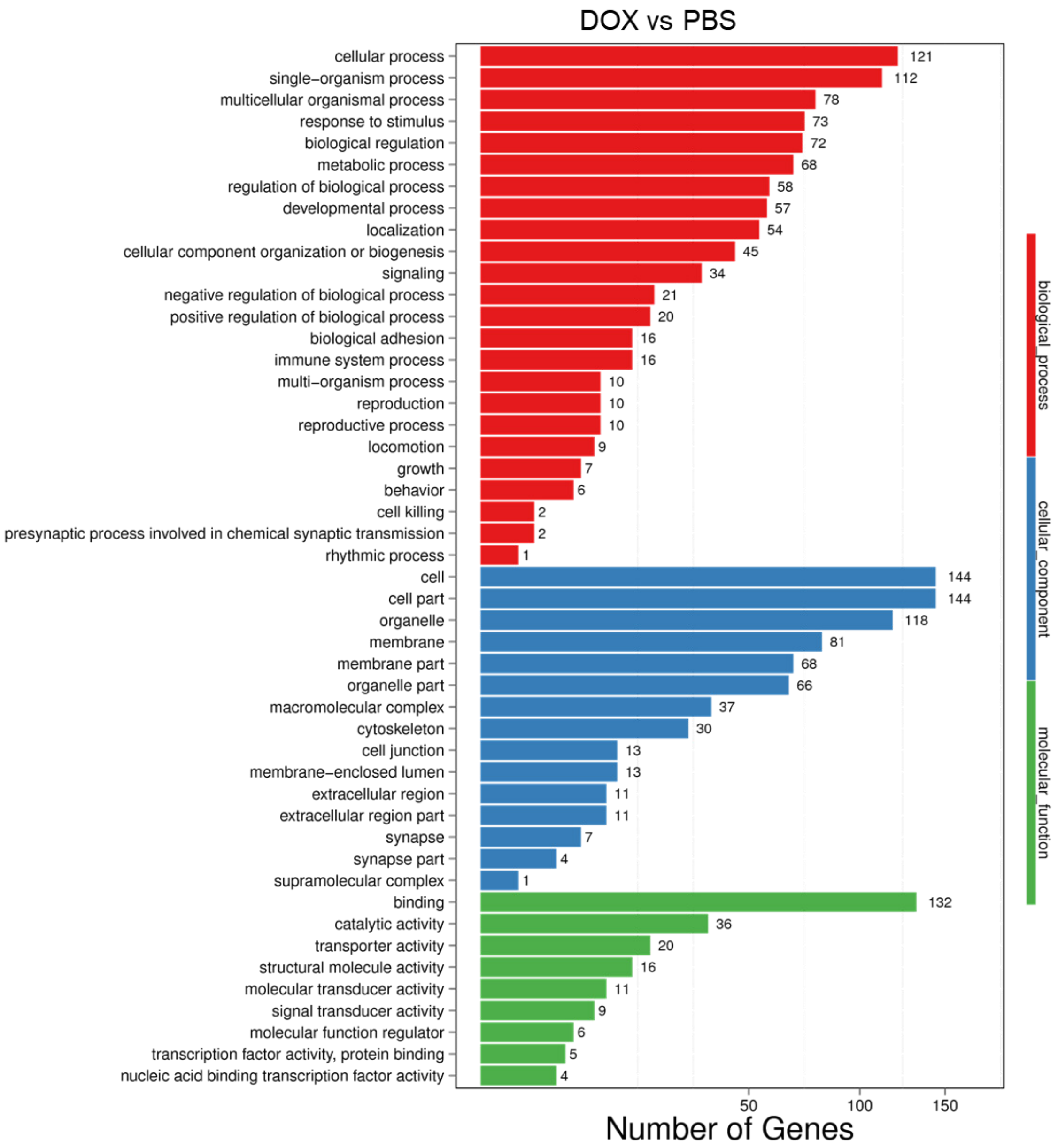

Figure S12. The globe gene changes between the mice receiving DOX and PBS treatments (n=2): DEGs number of the most enriched GO term. Up: log2FoldChange≥1, Padj≤0.05; Down: log2FoldChange≤-1, Padj≤0.05.

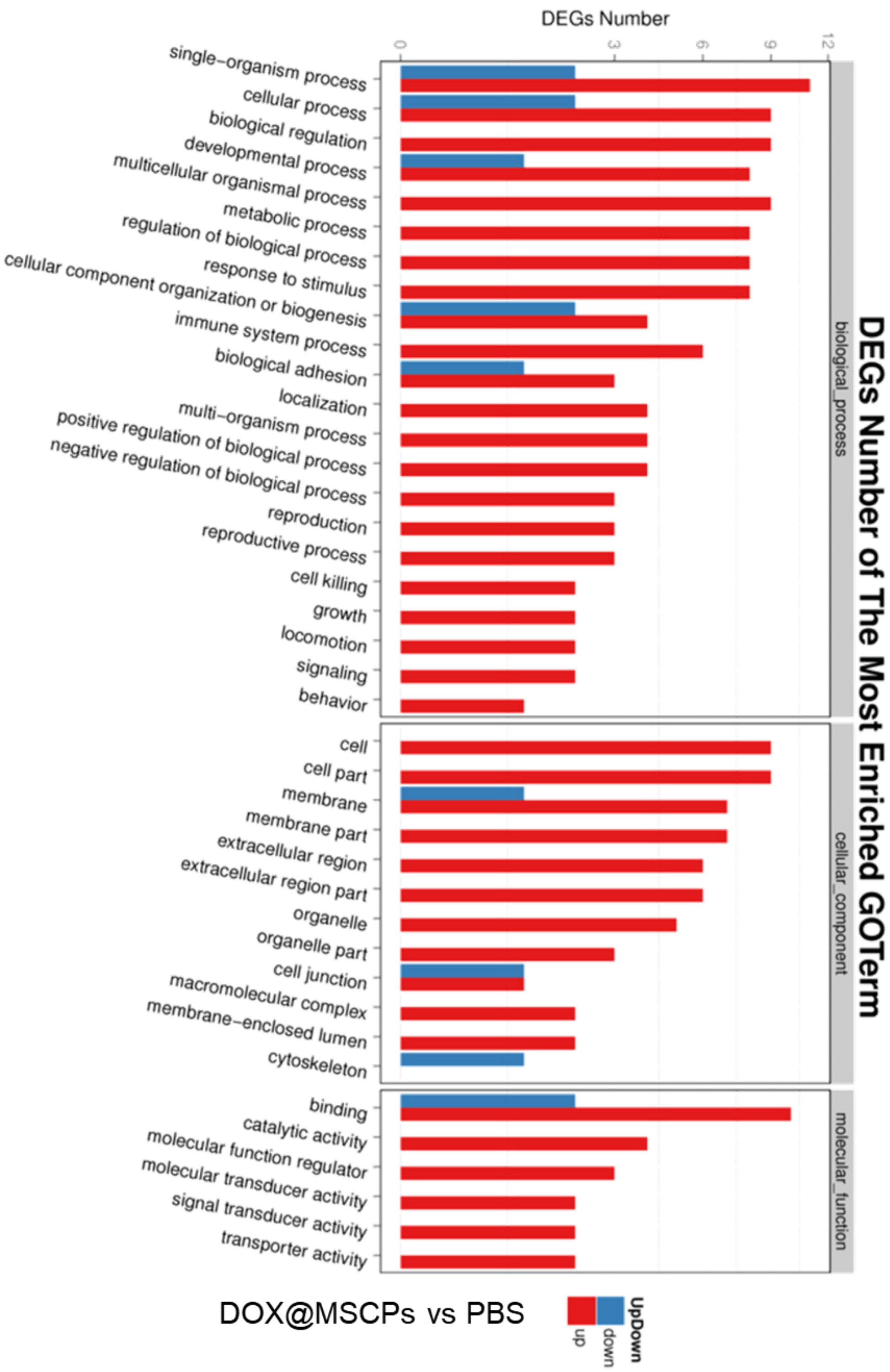

Figure S13. The globe gene changes between the mice receiving DOX@MSCPs and PBS treatments (n=2): Different expressed genes classified by biological functions, cellular component, and molecular functions based on GO annotation. Up: $\log_2$FoldChange≥1,

Padj≤0.05; Down: log_2FoldChange≤-1, Padj≤0.05.

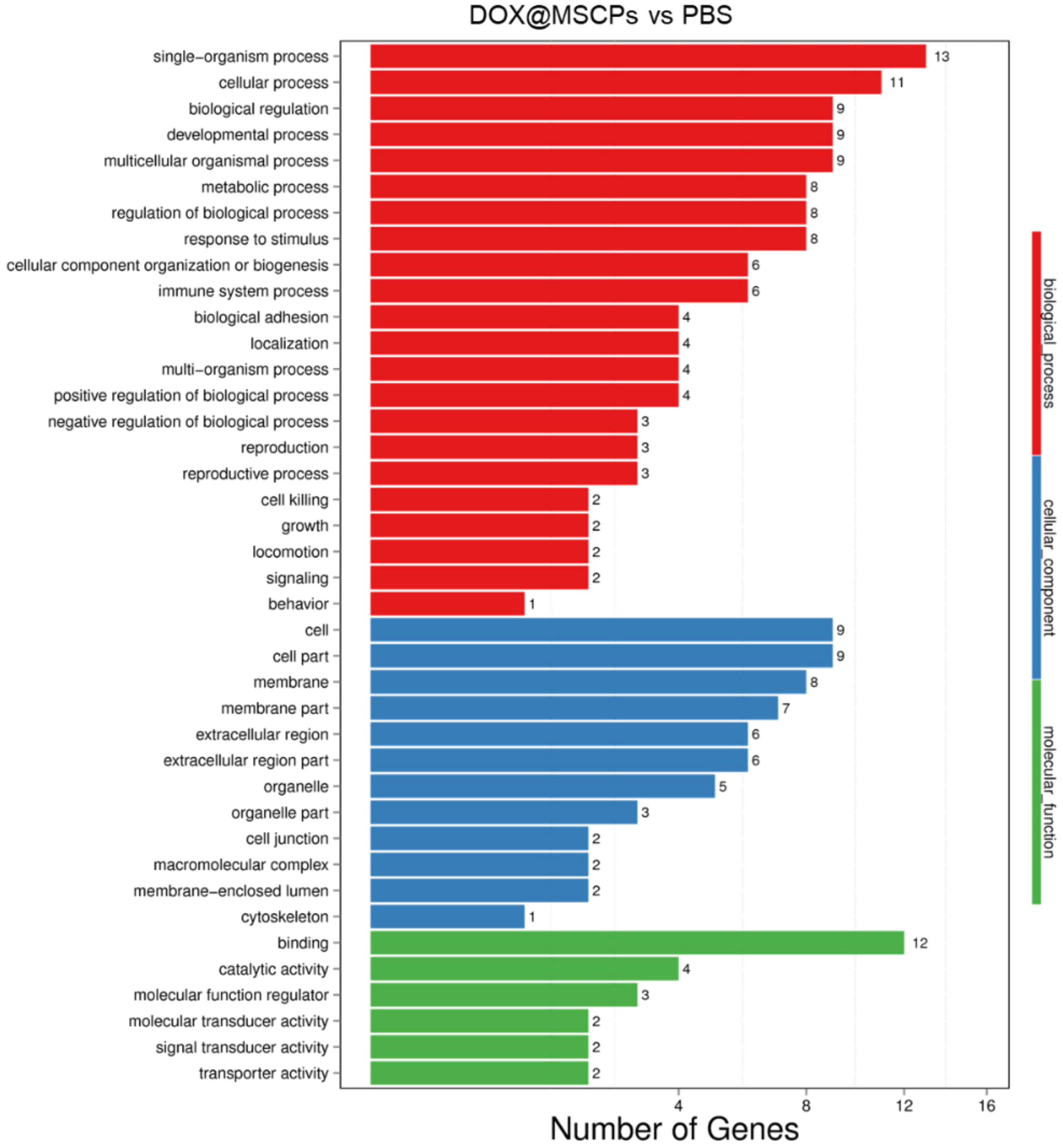

Figure S14. The globe gene changes between the mice receiving DOX@MSCPs and PBS treatments (n=2): DEGs number of the most enriched GO term. Up: log2FoldChange≥1, Padj≤0.05; Down: log2FoldChange≤-1, Padj≤0.05.

Table S1. The primer sequences used in q-PCR analysis.

Genes	Forward	Reverse
GAPDH	AAGGCTGTGGGCAAGG	TGGAGGAGTGGGTGTCG
P-gp	GGGAGCTTAACACCCGACTTA	GCCAAAATCACAAGGGTTAGCTT
Nanog	CCGAAGAATAGCAATGGTGTGACG	AGGAGAATTTGGCTGGAACTGC
Oct-3/4	TGGGTGGAGGAAGCTGACAA	TTTCTCTTTCGGGCCTGCAC
Nothch-3	GCAGCGATGGAATGGGTTTC	CTGCCAGGTTGGTGCAGATA

References

[1] J. Liu, Q. Li, J. Zhang, L. Huang, C. Qi, L. Xu, X. Liu, G. Wang, L. Wang, Z. Wang, Small **2017**, 13, 1602567.

[2] M. Fang, Y. Li, K. Huang, S. Qi, J. Zhang, W. Zgodzinski, M. Majewski, G. Wallner, S. Gozdz, P. Macek, A. Kowalik, M. Pasiarski, E. Grywalska, L. Vatan, N. Nagarsheth, W. Li, L. Zhao, I. Kryczek, G. Wang, Z. Wang, W. Zou, L. Wang, Cancer Res. **2017**, 77, 2735.

[3] J. Liu, X. Liu, Y. Yuan, Q. Li, B. Chang, L. Xu, B. Cai, C. Qi, C. Li, X. Jiang, G. Wang, Z. Wang, L. Wang, ACS Appl. Mater. Interfaces **2018**, 10, 26473.
